# Molecular and enzymatic insights into biocontrol-mediated resistance against Zucchini yellow mosaic virus in squash (*Cucurbita pepo* L.)

**DOI:** 10.1186/s12866-026-05161-x

**Published:** 2026-06-30

**Authors:** Radwa M. Shafie, Ahmed A. kheder, Mohamed A. Abou-Attia, Noher A. Mahmoud, Sawsan M. Saied, Amira A. Mazyad, Hayam S. Abdelkader

**Affiliations:** 1 Viruses and Phytoplasma Research Department, Plant Pathology Research Institute, Agricultural Research Center, Giza, Egypt; 2 Identification of Microorganisms and Biological Control Unit, Plant Pathology Research Institute, Agricultural Research Center (ARC), Giza, 12619 Egypt

**Keywords:** ZYMV, Antioxidant enzymes, Bio-Nagi, Bio-4, PR genes expression

## Abstract

Squash (*Cucurbita pepo* L.) is a major vegetable crop in Egypt and is highly vulnerable to *Zucchini yellow mosaic virus* (ZYMV), which causes severe mosaic, yellowing, and stunting symptoms. In this study, ZYMV was isolated from symptomatic squash plants in Kafr El-Sheikh Governorate and confirmed by RT-PCR and sequencing (GenBank Accession No. PV833355). The antiviral efficacy of two microbial biocontrol formulations-Bio-Nagi (*Trichoderma asperellum*) and Bio-4 (a *Bacillus* spp. consortium)-was assessed through foliar application and seed priming at pre-, concurrent-, and post-inoculation timings. Disease severity analyses showed that Bio-4 applied concurrently reduced symptom development by 62%, while Bio-Nagi pre-treatment resulted in a 48% reduction. Transmission electron microscopy revealed substantial protection of chloroplast and nuclear ultrastructure in Bio-4–treated plants compared with infected controls. Antioxidant enzyme activities (SOD and POD) increased by 1.8–2.4-fold following microbial treatments, indicating enhanced physiological defense responses. qRT-PCR profiling demonstrated strong differential induction of defense genes: PR1 was upregulated 5.6-fold in Bio-Nagi pre-treated plants, whereas PR3 and PR5 increased by 7–9-fold in Bio-4 concurrent and post-inoculation treatments. Overall, Bio-4 and Bio-Nagi mitigated ZYMV-associated symptoms and activated host defense pathways in squash. These findings underscore the importance of application timing and support microbial biocontrol agents as sustainable strategies for ZYMV management in squash production systems.

**Supplementary Information:**

The online version contains supplementary material available at 10.1186/s12866-026-05161-x.

## Background

Squash (*Cucurbita pepo* L.) is a major vegetable crop cultivated year-round in Egypt, with production occurring in greenhouses during winter and in open fields during summer. It is valued for its nutritional content-including proteins, soluble fibers, carbohydrates, and pro-vitamin A-and for its bioactive properties such as anti-inflammatory, antifungal, antibacterial, and anti-diabetic activities [12, 13, 18, 41]. Among the biotic stresses affecting squash productivity, plant viral diseases remain among the most destructive. *Zucchini yellow mosaic virus* (ZYMV), a member of the genus *Potyvirus*, causes severe mosaic, leaf deformation, chlorosis, and fruit malformation, resulting in significant yield and quality losses [4]. In Egypt, ZYMV has been widely reported using molecular diagnostic approaches [17, 27, 29, 33].

Microbial biocontrol agents have recently gained attention as sustainable alternatives to chemical antivirals, particularly due to their ability to activate plant immune responses. Several microbial genera-including *Bacillus*, *Paenibacillus*, *Streptomyces*, and *Trichoderma*-suppress viral infections by inducing systemic resistance, priming defense pathways, enhancing antioxidant capacity, and modulating SA-, JA-, and ET-mediated signaling [30, 42, 43]. *Bacillus* spp. can activate induced systemic resistance (ISR), produce lipopeptides and hydrolytic enzymes, stimulate ROS-scavenging responses, and modulate RNA interference pathways to limit viral replication [20, 39]. *Trichoderma* spp. similarly triggers ISR and SAR, enhance redox homeostasis, and have demonstrated antiviral activity against several plant viruses through immune priming [5, 30]. ISR-mediated protection against viral diseases is increasingly recognized, with studies reporting enhanced PR-gene activation and symptom reduction following microbial treatments [9, 37]. For instance, *Paenibacillus polymyxa* conferred up to 80% protection against ZYMV in squash [2].

Although microbial antagonists such as *Paenibacillus*, *Bacillus*, *Trichoderma*, and *Streptomyces* species have been explored for managing ZYMV, previous studies generally used single microbial isolates and focused mainly on symptom suppression or basic physiological responses. These limitations restrict understanding of how application timing influences the activation of antiviral defense pathways and how microbial consortia may enhance protection.

To address these gaps, the present study introduces two Egyptian microbial formulations currently under registration-Bio-Nagi (*Trichoderma asperellum*) and Bio-4 (a four-species *Bacillus* consortium)-which have not previously been evaluated against ZYMV. These formulations possess multiple potential antiviral mechanisms, including ISR/SAR activation, stimulation of antioxidant enzymes, and possible RNase-associated degradation of viral RNA, particularly within *Bacillus* consortia. The experimental design uniquely assesses both foliar and seed-priming applications across three inoculation timings (pre-, concurrent-, and post-infection), providing deeper insight into defense activation windows, mechanism specificity, and application strategy.

Overall, this study offers a comprehensive and novel assessment of microbial biocontrol strategies for mitigating ZYMV symptoms and enhancing host defense responses in squash, while clarifying how application timing influences antiviral effectiveness.

## Methods

### Plant material and sourcing

Seeds of *Cucurbita pepo* L. (cv. Eskandarani) used in the study were certified and taxonomically identified by experts from the Vegetable Crops Research Department, Plant Pathology Research Institute, Agricultural Research Center. The experimental work, including virus isolation and diagnostics, was conducted under prior approval from both the Vegetable Crops Research Department and the Virus and Phytoplasma Research Department, Plant Pathology Research Institute, Agricultural Research Center, in compliance with institutional ethical and biosafety regulations. All research complied with institutional, national, and international guidelines. No wild or endangered plant species were involved, and no CITES-listed taxa were used, so no additional permits were required.

### Virus isolation and propagation

Leaves of *Cucurbita pepo* cv. Eskandarani exhibiting typical ZYMV symptoms (mosaic, vein banding, yellowing, blisters, filiform growth, stunting, and deformation) were collected from a cultivated field in Baltim District, Kafr El-Sheikh Governorate, Egypt, following prior authorization from the field owner and approval from our institute. Symptomatic samples were tested by direct ELISA using Loewe antiserum (Germany), and ELISA-positive samples were mechanically inoculated onto squash plants under greenhouse conditions. The virus was biologically purified through three successive passages on *Chenopodium amaranticolor* and subsequently propagated on squash plants for use in downstream experiments. All experiments were conducted in a controlled greenhouse at the Plant Pathology Research Institute. Environmental conditions were maintained at 25 ± 2 °C during the day and 20 ± 2 °C at night, with 60–70% relative humidity and a 14 h light/10 h dark photoperiod. Plants were irrigated regularly and maintained under uniform management throughout the experiment.

### Ultrastructural studies

Transmission electron microscopy was performed on healthy, biocide-treated, and infected plants. Leaf pieces (2 × 2 mm) were fixed in 2% glutaraldehyde (0.1 M Na-cacodylate buffer, pH 7.2), post-fixed in 1% osmium tetroxide, dehydrated, and embedded in Spurr's resin. Ultrathin Sects. (90–100 nm) were cut with a diamond knife using a Leica EM-U ultramicrotome, placed on 400-mesh copper grids, and stained with 2% uranyl acetate (10 min) followed by lead citrate (5 min) [15, 38].

### RT-PCR detection and phylogenetic identification of ZYMV

Total RNA was extracted from healthy, infected, and treated squash plants using the RNeasy Plant Mini Kit (Qiagen, Germany). RT-PCR detection of ZYMV was performed using coat protein (CP)-specific primers ZYU-F and ZYD-R (Table S1) [35]. cDNA synthesis used Superscript II with primer ZYD-R. Thermal cycling consisted of 42 °C for 30 min, 94 °C for 1 min, followed by 35 cycles of 94 °C for 30 s, 65 °C for 30 s, 72 °C for 1 min, and a final extension at 72 °C for 5 min. PCR products were separated on 1% agarose gels stained with GelStar and visualized using a Gel Doc system (Bio-Rad) with a 100 bp ladder.

Phylogenetic analysis of the CP gene was conducted to compare the Egyptian isolate with global isolates. Sequences were aligned using ClustalW in CLC Genomics Workbench v25.0.2 with default gap penalties. Evolutionary relationships were inferred using the Neighbor-Joining method with the Kimura 2-parameter substitution model. Branch support was assessed with 1,000 bootstrap replicates. The dataset included 13 ZYMV CP sequences retrieved from GenBank representing geographically diverse regions (Egypt, Germany, China, Japan, Iran, Syria, India, Iraq, Kurdistan, South Korea, Australia, and Slovakia). Accession numbers are provided in the corresponding figure legend. RT-PCR served as a qualitative, end-point assay to confirm ZYMV presence. Because this method does not quantify viral load, results reflect viral detection rather than absolute accumulation.

### Quantitative real-time PCR (qRT-PCR) analysis of PR gene expression

Quantitative real-time PCR (qRT-PCR) was used to assess the expression of PR1, PR3, and PR5 genes in squash plants. Reactions were carried out using SYBR Green Master Mix (Applied Biosystems) on a StepOnePlus™ Real-Time PCR System. cDNA was synthesized using the RevertAid First Strand cDNA Synthesis Kit (Thermo Fisher Scientific). Primer sequences for all target genes (PR1, PR3, PR5), the reference gene (*β-actin*), and the ZYMV coat protein are listed in Table S1. Gene expression levels were normalized to *β-actin*, which served as the reference gene due to its stable expression across treatments (C_t_ variability < 1 C_t_). Relative transcript levels were calculated using the 2^ − ΔΔCt method. All qRT-PCR assays were conducted following essential MIQE guidelines. Primer specificity was verified by the presence of a single peak in melt-curve analysis and by a single expected amplicon on agarose gel electrophoresis. Primer efficiencies were calculated using a five-point, ten-fold serial dilution series of cDNA, with all efficiencies falling within the MIQE-accepted range (90–110%) and exhibiting R^2^ values ≥ 0.98. Although MIQE recommends multiple reference genes, only one validated housekeeping gene (*β-actin*) was used in this study; this limitation is acknowledged in the Discussion section. qRT-PCR analyses were performed using four biological replicates (*n* = 4) per treatment. For each biological replicate, three technical replicates were included. Technical replicates showed low variation, and mean C_t_ values were used for final expression calculations.

### Biocides and application methods

Accordingly, the following researchers assayed two biocides, Bio-Nagi and Bio-4 against ZYMV, to determine whether they were effective in managing virus infection. Bio-Nagi contains *Trichoderma asperellum* (2.5%, 10 × 10^6 spores/g), while Bio-4 is a mixture of four active bacterial agents: *Bacillus subtilis*, *Bacillus megaterium*, *Bacillus licheniformis*, and *Bacillus pumilus*. Thereafter, the two biocides were, respectively, applied at recommended dosages of 2.5 g/kg soaking and 2.5 g/L foliar spray and soil application. The application doses of Bio-Nagi and Bio-4 used in this study were determined based on preliminary greenhouse optimization experiments conducted prior to the trial, as well as manufacturer recommendations from the Identification of Microorganisms and Biological Control Unit (Plant Pathology Research Institute, ARC). These preliminary tests confirmed the chosen concentrations as the most effective and phytocompatible for squash plants.

#### Effects of foliar application

For pre-inoculation treatment, Bio-Nagi and Bio-4 were applied individually to the cotyledonary leaves of squash plants by foliar spraying. The plants were mechanically inoculated with ZYMV at 1 ml/plant after 48 h. The plants were first inoculated with ZYMV. In the post-inoculation treatment of squash cotyledonary leaves, treatments of Bio-Nagi and Bio-4 were given individually after 48 h. Control groups consisted of plants inoculated with ZYMV-infected or treated with a buffer solution pH 7.4 as healthy controls. Symptoms were observed during three weeks of post-inoculation.

#### Effects of seed treatment

Seeds of *Cucurbita pepo* were treated by soaking in 50 ml of each biocontrol agent for 6, 12, 18, and 24 h before planting. There were 20 seeds per treatment. Buffered solution (pH 7.4) was used as a control treatment, where seeds received this buffer treatment, and another set of seeds was untreated but infected. One week after germination, the cotyledonary leaves of the plants were inoculated with ZYMV. Recordings of symptoms were evaluated for three weeks post-inoculation. All experiments were conducted in a completely randomized block design with four replicates (5 seeds/pot). Both biocides are recently under registration and provided by the Unit of Identification of Microorganisms, Plant Pathology Institute, ARC, Egypt [3, 28].

### Evaluation of inhibition percentages and disease severity

The inhibition percentages and the disease severity were calculated to assess the treatment efficiency based on established methodologies. Percent inhibition was calculated using the following formula devised by Indira Devi et al., [26].

$$\mathrm{Inhibition}\left(\%\right)=\left(\frac{A-B}{A}\right)\times 100$$

where ***A*** represents the number of plants in the control group (untreated) and ***B*** is the number of plants in the treated and inoculated group. Each group has twenty squash plants.

### Disease severity assessment

Assessment of the severity of disease caused by ZYMV was done 21 days post inoculation. The assessment of the severity of symptoms was done on a scale ranging from 0 to 5 as described by Sofy et al. [40]. The disease severity percentage was calculated using the formula:

$$\mathrm{DS}\left(\%\right)=\frac{\sum\left(\text{No. of plants in each grade}\times\text{disease grade}\right)}{\text{Total number of plants}\times\text{highest grade}}$$

This formula calculates the total severity grades for all plants and normalizes the value based on the maximum possible severity grade. Each treatment group consisted of 20 squash plants.

### Effect of Bio-Nagi and Bio-4 on enzyme activity

To evaluate the impact of Bio-Nagi and Bio-4 on enzyme activity, twenty squash plant treated with these biocides were analyzed pre- concurrent and post-ZYMV inoculation. Enzyme extraction was conducted following the protocol described by Anand et al. [8]. Leaf tissues from treated squash plants were homogenized in liquid nitrogen and one gram of the powdered leaf sample was extracted using 2 ml of 0.1 M sodium phosphate buffer (pH 7.0) at 4ºC. Peroxidase activity (POD) was assessed using methods described by Hartee [24], Hammerschmidt et al. [23], and Allam and Hollis [6]. Superoxide dismutase activity (SOD) was determined according to the protocol described by Giannopolitis and Ries [19].

### Data analysis

All statistical analyses were performed using StatGraphics Centurion XVI (StatPoint Technologies, USA) and CoStat software (version 6.4, Cohort Software, USA). Depending on the experimental design, data were analyzed using one-way or two-way ANOVA. Depending on the dataset, ANOVA was followed by either the Least Significant Difference (LSD) test or Tukey’s Honest Significant Difference (HSD) test at *p* < 0.05 to determine differences among treatment means. For disease severity, inhibition percentage, and enzyme activity data, two-way ANOVA was performed with treatment and application timing as fixed factors, followed by the Least Significant Difference (LSD) test for multiple comparisons at *p* < 0.05. For PR1, PR3, and PR5 gene expression data obtained from qRT-PCR, one-way ANOVA was used, and post hoc comparisons were conducted using Tukey’s Honestly Significant Difference (HSD) test (*p* < 0.05) to correct for multiple comparisons. Each treatment consisted of four biological replicates (*n* = 4), and all qRT-PCR reactions were performed using three technical replicates per biological sample. All quantitative data are presented as mean ± standard deviation (SD). The corresponding figure legends specify the statistical tests applied, replicate structure, units, and the criteria used to determine significance.

## Results

### Virus isolation and molecular identification

**Fig. 1:**
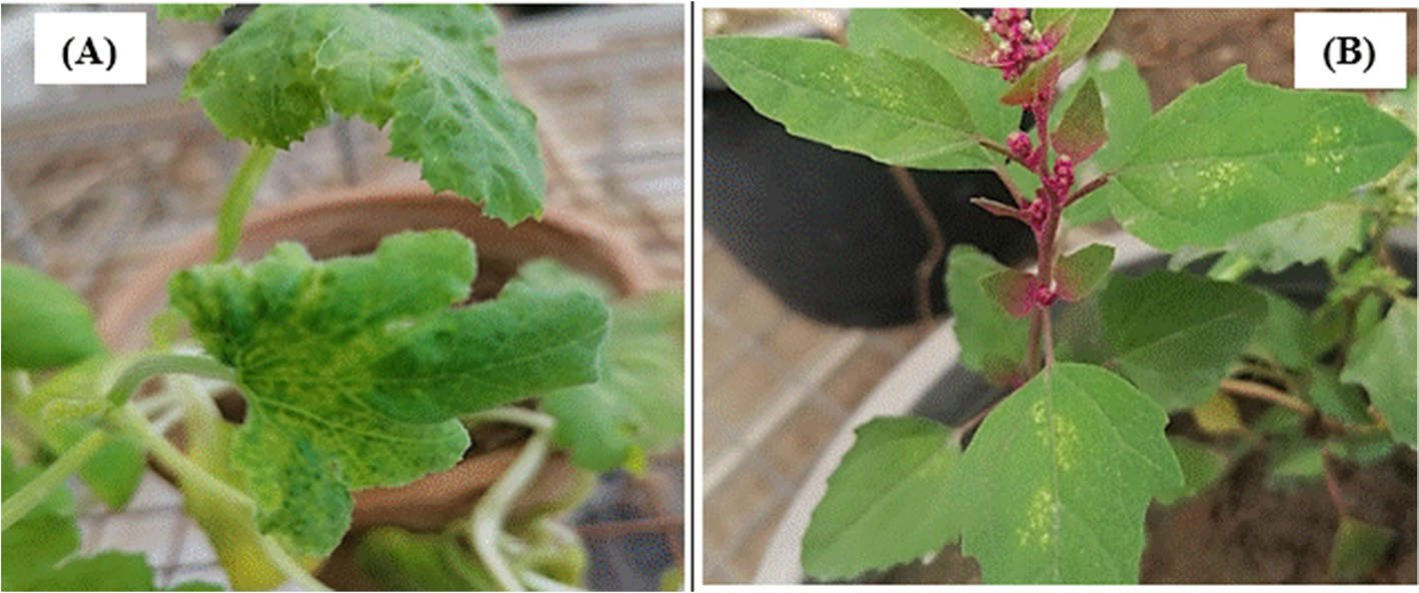
**A** Typical mosaic symptoms of ZYMV on squash plants. **B** ZYMV causes chlorotic (yellow) spots on *Chenopodium amaranticolor*

**Fig. 2:**
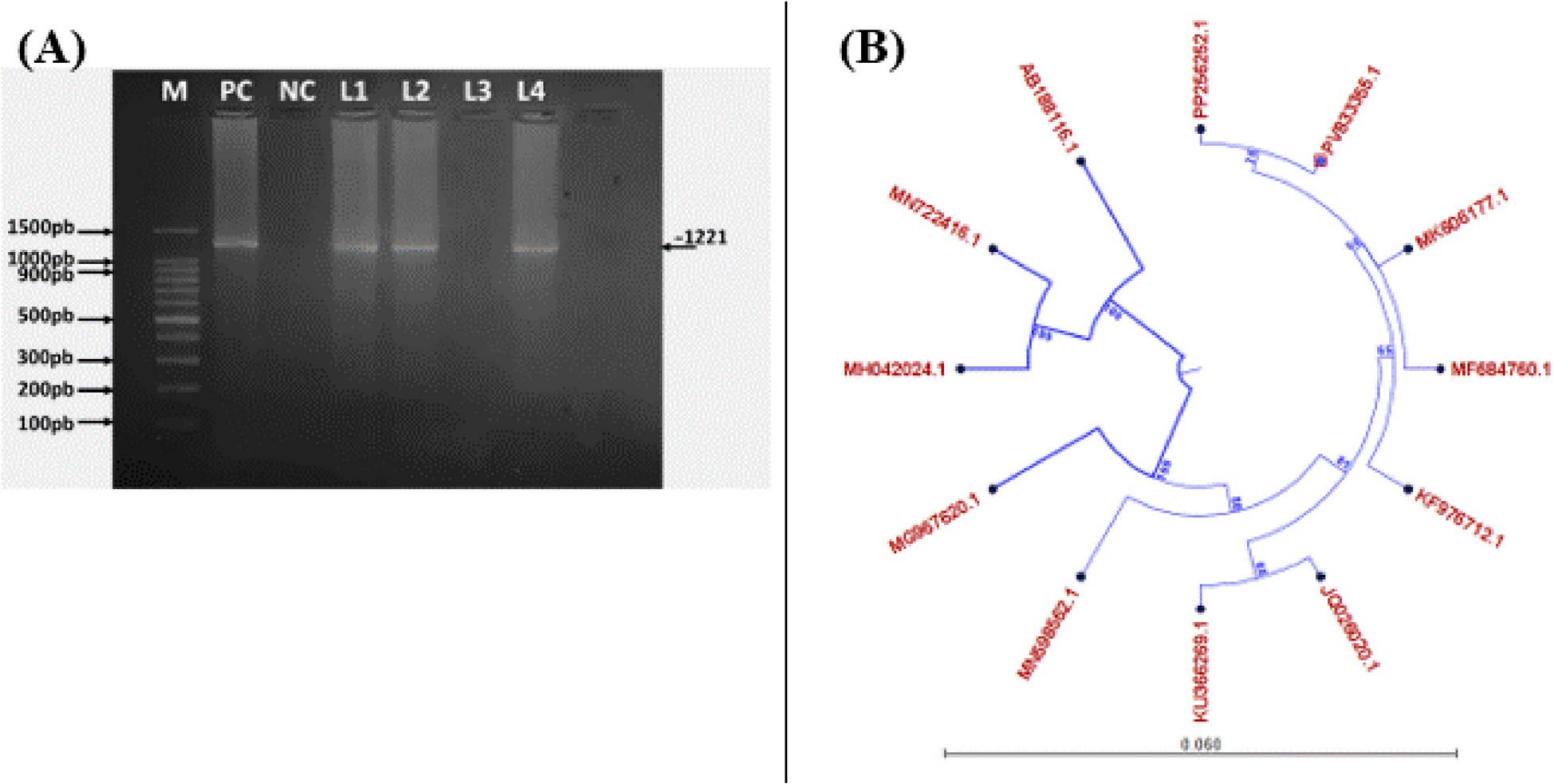
**A** RT-PCR amplification of the Zucchini yellow mosaic virus (ZYMV) coat protein (CP) gene showing a specific ~ 1221 bp amplicon in infected squash plants. Lane M: 100 bp DNA ladder; PC: positive control; NC: negative control; L1, L2, L4: infected samples; L3: healthy plant sample.** B** Phylogenetic tree of ZYMV isolates based on CP gene sequences. Multiple sequence alignment was performed using ClustalW, and the tree was constructed using the Neighbor-Joining (NJ) method with the Kimura 2-parameter substitution model. Branch support was assessed using 1,000 bootstrap replicates, and only values > 50% are shown. The dataset included 13 ZYMV isolates representing diverse geographical regions: Egypt (PV833355.1, this study), Syria (MK606177.1), China (MN722416.1), Iran (KU366269.1), Japan (AB188116.1), Australia (MN598562.1), Germany (PP256252.1), Kurdistan (MF684760.1), Iraq (JQ026020.1), India (MG967620.1), South Korea (MH042024.1), and Slovakia (KF976712.1). ZYMV-EG isolate (PV833355.1) clusters most closely with the German isolate (PP256252.1) with 99.67% nucleotide identity. The scale bar indicates the number of nucleotide substitutions per site. Full-length, uncropped gel images are provided in Additional file S1

ZYMV was isolated from *Cucurbita pepo* cv. Eskandarani showing typical viral symptoms (mosaic, vein banding, yellowing, leaf deformation, and fruit blisters) in Giza, Egypt. The virus was biologically purified via local lesion assay on *Chenopodium amaranticolor* and propagated on squash (Fig. 1). Identification was based on symptomatology and RT-PCR amplification of a 1221 bp fragment of the coat protein gene in infected samples and positive control, with no amplification in healthy or negative controls (Fig. 2A). Sequence and phylogenetic analysis of the CP gene (isolate PV833355.1) using CLC Genomics Workbench v25.0.2 revealed 99.67% identity with the German isolate PP256252.1 and strong clustering supported by a 74% bootstrap value (Fig. 2B), indicating close genetic relatedness.

### Ultrastructural alterations in ZYMV-infected squash leaves

**Fig. 3:**
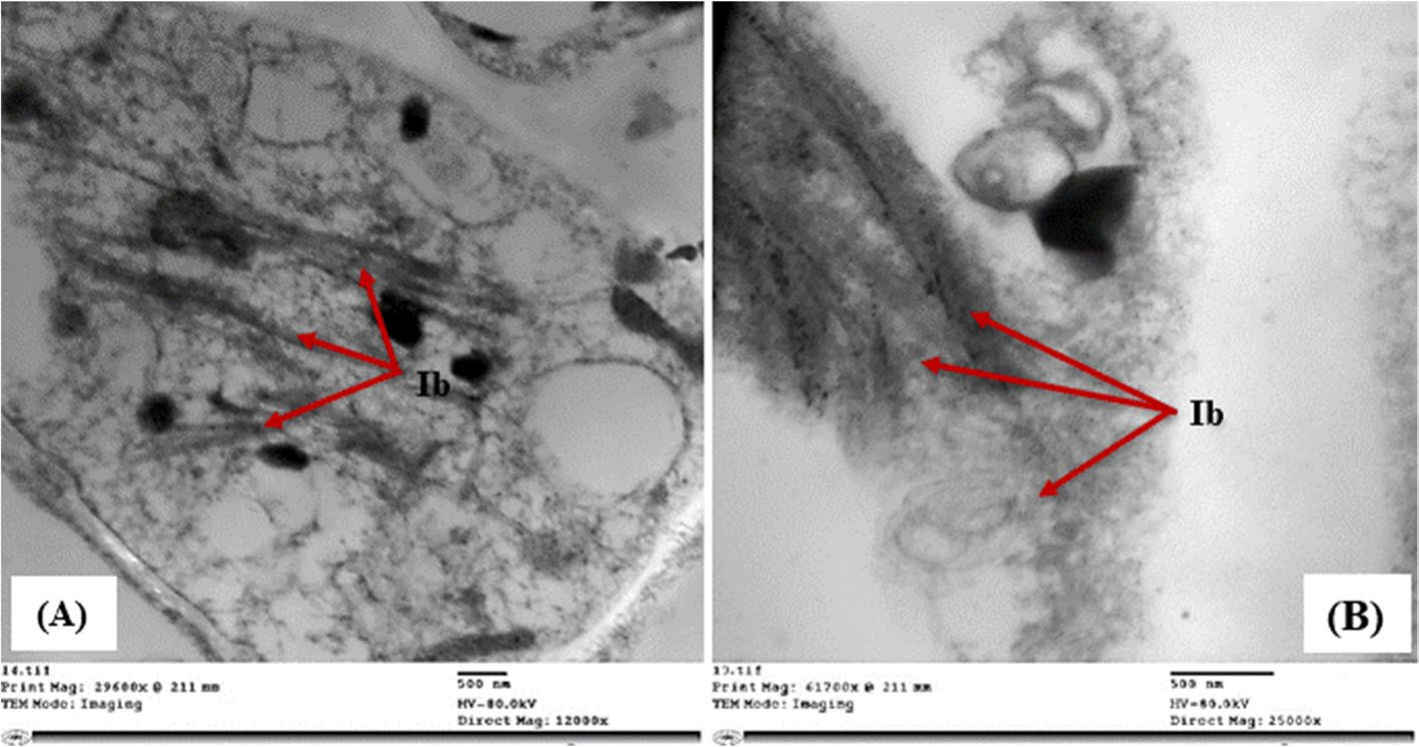
Transmission electron micrographs showing the ultrastructure of ZYMV-infected squash leaf cells. **A–B** Cytoplasmic inclusion bodies characteristic of potyvirus infection are visible as elongated or filamentous electron-dense structures (red arrows). Images were obtained from three independent biological samples. Scale bars = 500 nm. Magnifications: (**A**) 29,600 ×; (**B**) 61,700 ×

Electron micrographs (Fig. 3) revealed dense, filamentous inclusion bodies within the cytoplasm of infected cells, typical of potyviruses. Their cylindrical morphology confirms active ZYMV replication and particle assembly in squash leaf tissues.

### Efficacy of Bio-Nagi and Bio-4 against ZYMV symptoms in squash

**Fig. 4:**
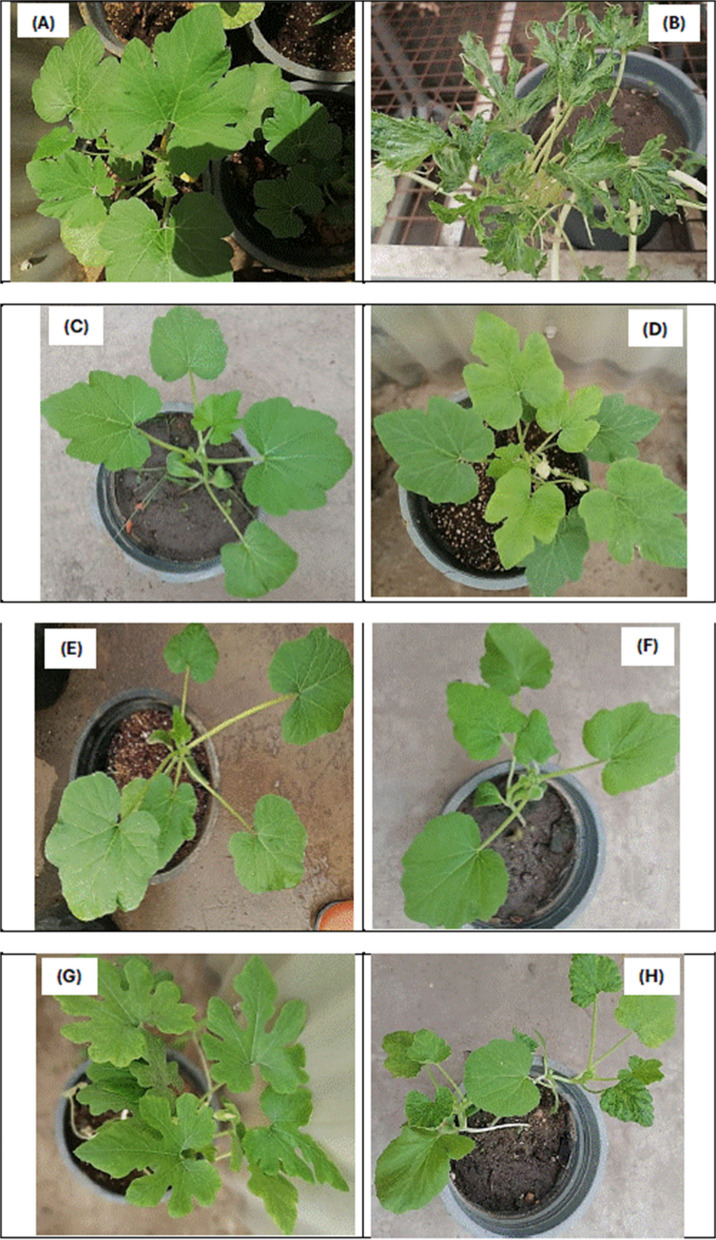
Effects of Bio-Nagi and Bio-4 Treatments on Squash Plants Inoculated with ZYMV. **A** Healthy squash plants show no disease symptoms, (**B**): ZYMV-infected plants display severe symptoms such as stunted growth, yellowing, and leaf deformation. **C** Bio-4 applied 48 h before ZYMV inoculation effectively reduces disease symptoms, indicating enhanced resistance. **D** Concurrent application of Bio-4 with ZYMV inoculation also provides moderate protection, though less effective than pre-inoculation treatment. **E** Post-inoculation application of Bio-4 shows diminished efficacy, with more pronounced disease symptoms. **F** Bio-Nagi applied 48 h before ZYMV inoculation moderately reduces disease symptoms. **G** Bio-Nagi foliar application concurrently with ZYMV inoculation offering some protection. **H** Post-inoculation application of Bio-Nagi results in more severe symptoms, indicating limited effectiveness. All photographs were taken on the same day inside the greenhouse under identical natural lighting conditions using the same camera settings

The timing of biocontrol application significantly influenced symptom severity in ZYMV-infected squash plants. Pre-treatment with Bio-4, applied 48 h before virus inoculation, offered the greatest protection, followed by concurrent treatment, while post-infection application was least effective. Bio-Nagi showed a similar trend but was generally less effective than Bio-4. Figure 4 presents visual comparisons of treated and untreated plants. Healthy controls exhibited normal growth, while untreated infected plants showed pronounced stunting and leaf deformation. Treated plants displayed varying degrees of symptom reduction depending on the timing and product applied.

### Efficacy of Bio-Nagi vs. Bio-4 in controlling ZYMV

**Fig. 5:**
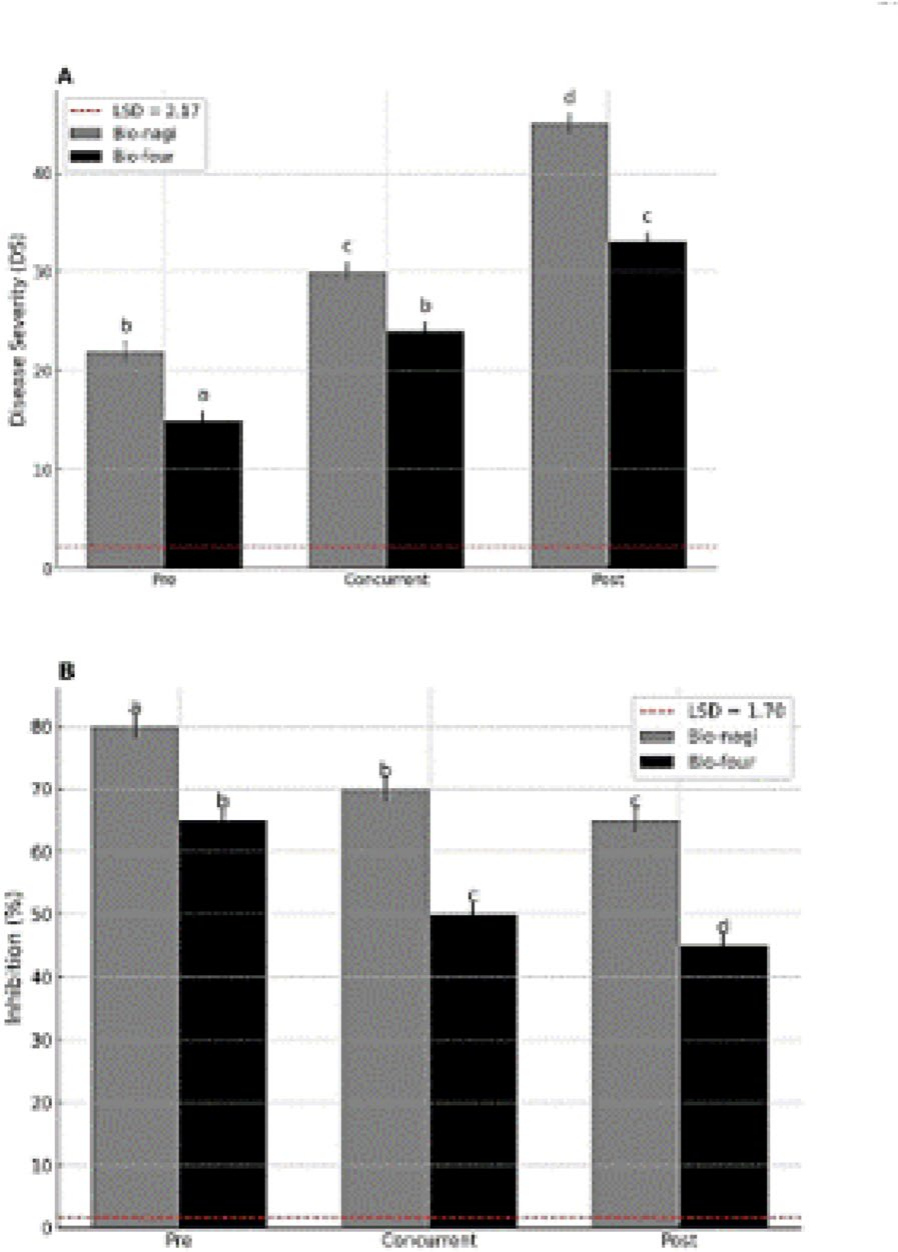
**A** Disease severity (DS, %) of squash plants treated with Bio-Nagi and Bio-4 at three application timings: pre-inoculation, concurrent with inoculation, and post-inoculation with ZYMV. **B** Inhibition percentage (%) corresponding to each treatment and timing. Data represent the mean ± standard deviation (SD) of four biological replicates (*n* = 4). Statistical analysis was performed using two-way ANOVA, and mean separation was conducted using the Least Significant Difference (LSD) test at *p* < 0.05. The red dashed lines indicate the LSD values (2.17 for DS and 1.70 for inhibition). Bars sharing the same letter do not differ significantly according to LSD. Error bars represent SD

Bio-Nagi pre-treatment reduced disease severity (DS) values (21.39–22.91) with inhibition rates of 55–65%, showing greater effectiveness than concurrent or post-infection applications. Concurrent treatment showed moderate DS (29.15–31.53) and inhibition (45–55%), while post-inoculation treatment was least effective (DS: 44.22–47.52; inhibition: 30–60%). Bio-4 consistently outperformed Bio-Nagi. Pre-treatment resulted in the lowest DS values (14.21–15.89) and highest inhibition (70–90%). Concurrent and post-infection treatments remained effective but showed slightly higher DS and lower inhibition rates. Overall, pre-treatment was most effective for both agents. Bio-4 significantly outperformed Bio-Nagi at all time points (*p* < 0.05), with inhibition rates of 80%, 70%, and 65% compared to 60%, 50%, and 45% for Bio-Nagi (Fig. 5B), confirming Bio-4’s superior antiviral efficacy.

### Ultrastructural observations

**Fig. 6:**
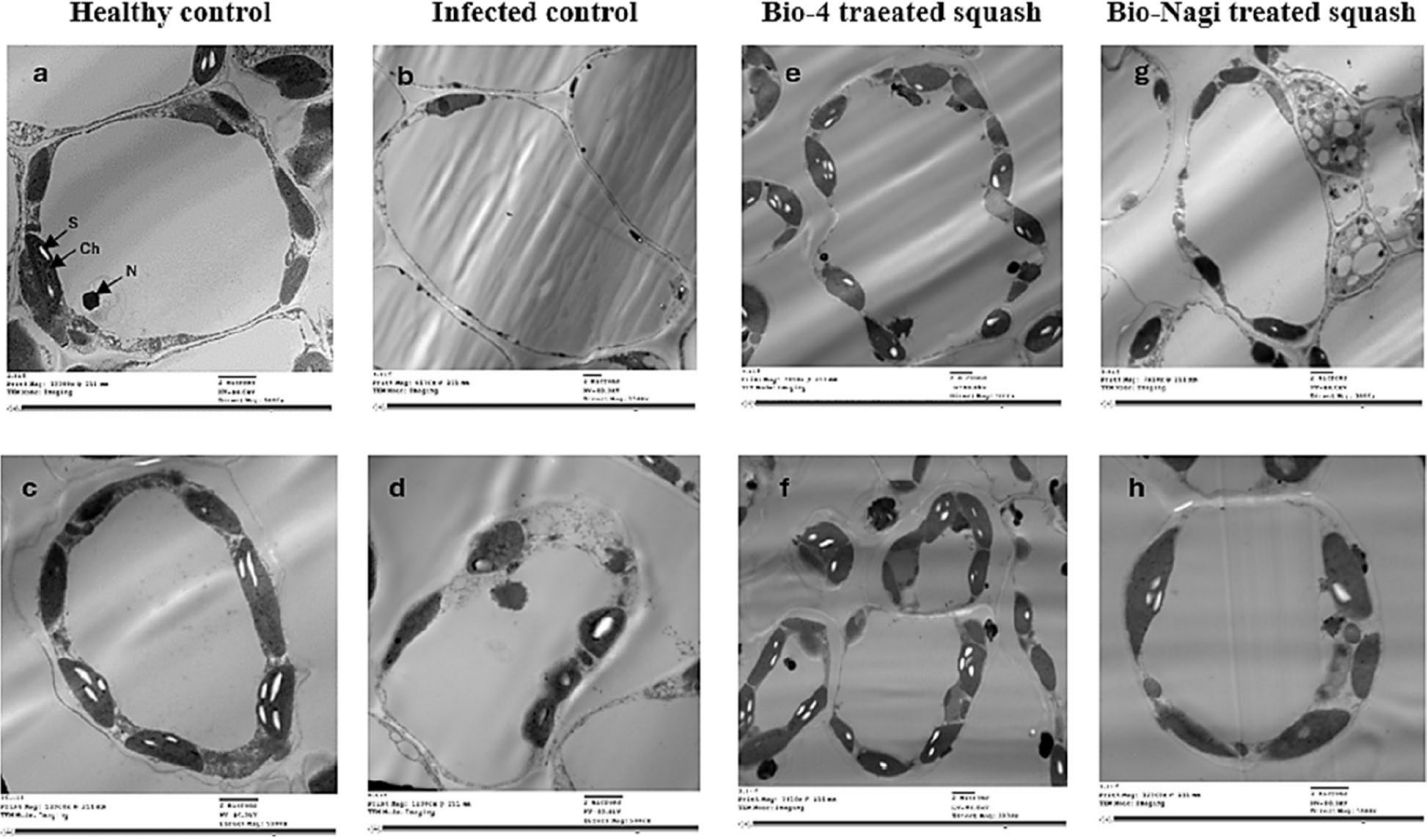
Transmission electron micrographs of squash leaf tissues from: (**a**, **c**) healthy control plants, (**b**, **d**) ZYMV-infected control plants, (**e**, **f**) Bio-4–treated plants, and (**g**, **h**) Bio-Nagi–treated plants. Healthy cells show intact chloroplasts (Ch), nucleus (N), and starch granules (S). Infected controls exhibit chloroplast disorganization, nuclear deterioration, plasmolysis, and thickened cell walls. Bio-4 and Bio-Nagi treatments show partial restoration of cellular integrity, with improved chloroplast and nuclear structure and reduced plasmolysis relative to infected controls. Images were obtained from three independent biological samples. Scale bar = 500 nm

The electron micrographs presented provide detailed insights into the ultrastructural changes in squash leaves under different treatments (Bio-4 treated squash, Bio-Nagi treated squash, healthy control and infected control) (Fig. 6). The cellular structures in the healthy control squash leaves (a and c) are well-preserved. Intact chloroplasts (Ch) with well-defined thylakoid membranes are visible, along with a clearly defined nucleus (N) and starch granules (S). The cell walls are uniformly thin, and the organelles are evenly distributed within the cytoplasm, indicating a healthy cellular state. The squash leaves infected with ZYMV (b and d) show significant ultrastructural alterations. The chloroplasts are disorganized and show signs of degradation. The nuclei appear damaged or fragmented, indicating compromised cellular integrity. The overall cellular structure is disrupted, with evidence of plasmolysis and cell wall thickening.

The Bio-Four treated squash leaves (e and f) exhibit improved cellular preservation compared to the infected control. The chloroplasts and nuclei show reduced degradation, suggesting partial recovery or protection conferred by the treatment. The cell walls are more intact, with fewer signs of plasmolysis and structural damage.

The Bio-Nagi treated squash leaves (g and h) also show improved cellular structures compared to the infected control. There is better preservation of chloroplasts and nuclei, although some signs of cellular stress remain. The cell walls are more intact compared to the infected control, indicating some level of protection and recovery due to the Bio-Nagi treatment.

### Evaluation of ZYMV inhibition in squash seeds treated with Bio-Nagi and Bio-4

**Fig. 7:**
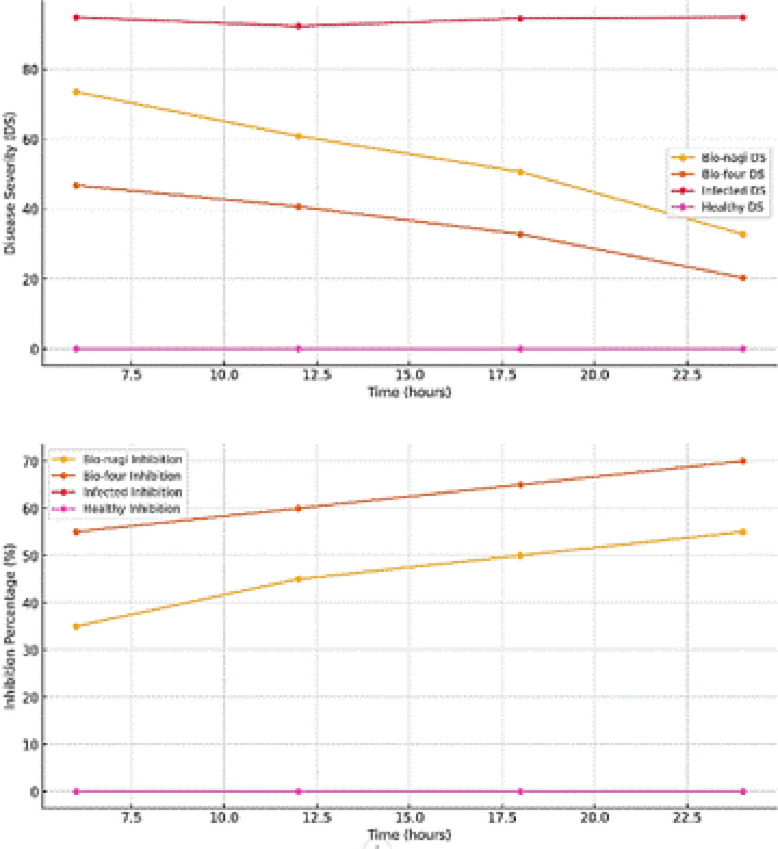
Disease severity (DS, %) and inhibition percentage (%) of squash plants subjected to seed treatments with Bio-Nagi and Bio-4 over a 24-h time course, compared with infected and healthy controls. The top panel presents DS values, showing that Bio-4-treated plants consistently exhibited the lowest disease severity, particularly at later time points, while infected controls maintained the highest DS and healthy controls remained symptom-free (DS = 0). The bottom panel displays inhibition percentage, with Bio-4 showing the greatest inhibition (approaching 100% at 24 h), followed by Bio-Nagi. As expected, infected controls exhibited 0% inhibition. Data represent the mean ± standard deviation (SD) of four biological replicates (*n* = 4). Statistical analysis was performed using two-way ANOVA, and differences among means were evaluated using the Least Significant Difference (LSD) test at *p* < 0.05. Error bars represent SD, and bars or time points sharing the same letter are not significantly different

The study investigated the effects of soaking squash seeds in Bio-Nagi and Bio-4 treatments on the infectivity of ZYMV over different time intervals. The number of infected plants, disease severity (DS) percentage, and inhibition percentage were analyzed for the various treatment groups (Fig. 7). The Bio-Nagi treatment exhibited the highest initial number of infected plants (13) and DS (72.83%) when seeds were soaked for 6 h. However, these values steadily declined over time, reaching 9 infected plants and 32.78% DS by 24 h. In contrast, soaking seeds in Bio-Nagi for 12 h resulted in 45% inhibition and 60.85% DS. Soaking squash seeds in the Bio-4 treatment showed more promising results. At 6 h, Bio-4 had a lower initial number of infected plants (9) and DS (46.73%) compared to Bio-Nagi. These values continued to decrease dramatically, ultimately reaching 6 infected plants and 20.33% DS by the 24-h mark, with an impressive 70% inhibition rate. The Infected control group maintained consistently high levels, with 20 infected plants and around 94–95% DS throughout the study period. Conversely, healthy control showed no infected plants and 0% DS across all time points. Compared to the infected control, where the inhibition percentage was 0% and the disease severity was 94.83%, the Bio-Nagi treatment at 24 h achieved a 55% inhibition rate and 32.87% DS. The Bio-4 treatment, on the other hand, gave the highest significant inhibition percentages of 70% and 20.33% DS when seeds were soaked for 24 h.

A two-way ANOVA was performed with treatment and time as the independent variables and DS or inhibition percentage as the dependent variables. The analysis revealed a significant interaction effect between treatment and time for both DS (*p* < 0.001) and inhibition percentage (*p* < 0.001), indicating that the effect of the treatments on these outcomes varied over time. Post-hoc pairwise comparisons further confirmed that the Bio-4 treatment resulted in significantly lower DS (*p* < 0.01) and higher inhibition percentage (*p* < 0.01) compared to the Bio-Nagi treatment at the later time points (18 and 24 h). The consistent 0% DS and inhibition for Healthy control confirmed that this group was not impacted by the disease or treatments, as expected. These results suggest that the Bio-4 treatment was the most effective in reducing disease severity and promoting inhibition compared to the other treatments tested in this study. The comparison of Disease Severity (DS) at 24 h post-treatment indicates that the difference between the Bio-Nagi and Bio-4 treatments is not statistically significant. While Bio-4 shows a tendency towards greater efficacy in reducing DS compared to Bio-Nagi, this difference does not reach statistical significance. On the other hand, the difference in DS between the Infected and Healthy control groups is statistically significant, reflecting the expected higher DS in the Infected group. This suggests that the Bio-4 treatment, despite appearing more effective, does not significantly outperform Bio-Nagi in this study, whereas infection markedly increases DS compared to the healthy condition.

### The effect of foliar application of Bio-Nagi and Bio-4 on enzyme activity in squash plants

The study investigated the effect of Bio-Nagi and Bio-4 foliar applications on SOD and POD enzyme activities in squash plants 48 h pre- concurrent and post virus inoculation. The results demonstrate significant physiological responses in enzyme activities following these treatments.

**Fig. 8:**
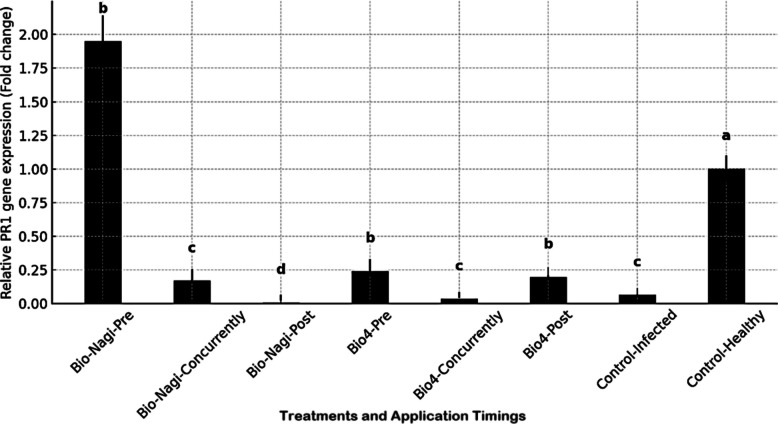
Relative expression of PR1 gene in squash plants infected with *Zucchini yellow mosaic virus* (ZYMV) and treated with Bio-Nagi and Bio-4 at different application timings. Treatments include pre-inoculation, concurrent with inoculation, and post-inoculation applications. Expression levels were normalized to actin as a housekeeping gene and analyzed using the 2^(-ΔΔC_t_) method. Data are presented as mean fold change relative to the healthy control. Error bars indicate ± standard error (SE). Different letters above bars denote statistically significant differences (*p* < 0.05) based on Tukey's HSD test. (Additional file S1)

For SOD enzyme activity, both Bio-Nagi and Bio-4 significantly increased the activity levels compared to the infected and healthy controls. Bio-4 exhibited the highest SOD activity, followed by Bio-Nagi, while the infected and healthy controls showing the lowest activities. A notable decrease in SOD activity was observed from the pre-treatment phase to the post-treatment phase for both Bio-Nagi and Bio-4, indicating a transient response to the initial treatment (Table 1 and Fig. 8A).

**Table 1:** Superoxide dismutase (SOD) activity (U g⁻^1^ fresh weight) in squash plants treated with Bio-Nagi and Bio-4 at three application timings relative to ZYMV inoculation

Treatment	Pre (Mean ± SD)	Concurrent (Mean ± SD)	Post (Mean ± SD)	Pre *vs* Concurrent		Pre *vs* Post		Concurrent *vs* Post	
				Paired-*t*-test	*p*-value	Paired-*t*-test	*p*-value	Paired-*t*-test	*p*-value
Bio-Nagi	131.87 ± 4.12	129.90 ± 3.87	125.58 ± 4.33	48.66	0.0004	112.23	0.00008	95.80	0.0001
Bio-4	173.44 ± 5.21	161.25 ± 4.97	140.43 ± 5.42	276.29	0.00001	935.65	0.000001	397.49	0.000006
Infected C	75.11 ± 2.11	75.11 ± 2.11	75.11 ± 2.11	0.00	1.0000	0.00	1.0000	0.00	1.0000
Healthy C	60.40 ± 1.95	60.40 ± 1.95	60.40 ± 1.95	0.00	1.0000	0.00	1.0000	0.00	1.0000

Pre = 48 h before inoculation; Concurrent = during inoculation; Post = 48 h after inoculation

Regarding POD enzyme activity, Bio-4 also showed the highest activity levels, followed by Bio-Nagi, while the infected and healthy controls exhibiting the lowest levels. Both Bio-Nagi and Bio-4 treatments resulted in a significant increase in POD activity compared to controls, with a similar decreasing trend over time observed in both treatments (Table 2 and Fig. 8B).

**Table 2:** Peroxidase (POD) activity (U g⁻^1^ fresh weight) in squash plants treated with Bio-Nagi and Bio-4 at three application timings relative to ZYMV inoculation

Treatment	Pre (Mean ± SD)	Concurrent (Mean ± SD)	Post (Mean ± SD)	Pre *vs* Concurrent		Pre *vs* Post		Concurrent *vs* Post	
				Paired-*t*-test	*p*-value	Paired-*t*-test	*p-*value	Paired-*t*-test	*p*-value
Bio-Nagi	2.775 ± 0.105	2.412 ± 0.098	2.270 ± 0.113	174.38	0.00003	200.67,	0.00002	142.00,	0.00005
Bio-4	3.950 ± 0.132	3.514 ± 0.123	3.460 ± 0.141	251.72	0.00002	277.61	0.00001	18.84	0.0028
Infected C	1.024 ± 0.078	2.683 ± 0.091	2.649 ± 0.097	33.43	0.0009	25.18	0.0015	3.33	0.1
Healthy C	0.619 ± 0.047	0.619 ± 0.047	0.619 ± 0.047	0.00	1.0000	0.00	1.0000	0.00	1.0000

Pre = 48 h before inoculation; Concurrent = during inoculation; Post = 48 h after inoculation.; Mean ± SD = Mean ± Standard Deviation; *p*-value = probability value from paired *t*-test indicating statistical significance. Statistical comparisons between timings were conducted using paired *t*-tests

Statistical analysis showed significant differences in enzyme activities across pre-, concurrent-, and post-inoculation treatments (*p* < 0.05). The Least Significant Difference (LSD) for SOD was calculated as 143.70 and for POD as 4.27.

These results suggest that Bio-Nagi and Bio-4 are effective in inducing a defense response in squash plants, as evidenced by the increased activities of SOD and POD enzymes. The transient nature of this response, with a peak at the initial stages followed by a decline, may reflect an adaptive mechanism to the induced stress by these treatments.

### The relative expression levels of the PR1 gene

The relative expression levels of the PR1 gene in squash plants infected with Zucchini yellow mosaic virus (ZYMV) and treated with Bio-Nagi and Bio-4 at different application timings (pre-inoculation, concurrently with inoculation, and post-inoculation) were assessed using the 2^(-ΔΔCt) method, normalized against a healthy control. Significant differences (*p* < 0.05) were observed among the treatments. The highest PR1 gene expression occurred in plants treated with Bio-Nagi prior to virus inoculation ("Bio-Nagi-Pre"), exhibiting approximately a two-fold increase relative to the healthy control. Moderate gene expression was recorded in plants treated with Bio4 prior to inoculation ("Bio4-Pre") and Bio4 post-inoculation ("Bio4-Post"), which showed significantly lower expression than the "Bio-Nagi-Pre" treatment (Table 3 and Fig. 8). Treatments applied concurrently with virus inoculation ("Bio-Nagi-Concurrently" and "Bio4-Concurrently"), as well as Bio-Nagi post-inoculation ("Bio-Nagi-Post") and the infected untreated control, exhibited significantly reduced PR1 gene expression, indicating minimal defense activation. Thus, timing and type of biocontrol application substantially influenced the host defense gene response, with pre-inoculation application of Bio-Nagi showing the most pronounced protective gene expression response.

**Table 3:** ΔC_t_, ΔΔC_t_, and relative fold change (2⁻ΔΔC_t_) of PR1 gene expression in squash plants treated with Bio-Nagi and Bio-4 at different application timings relative to ZYMV inoculation

Treatment	C_t_-PR1	C_t_-Actin	ΔC_t_	ΔΔC_t_	Fold Change (2^ ^−ΔΔC^_t_)
Bio-Nagi-Pre	36.5	33.536	2.964	−0.961	1.95 ± 0.12^b^
Bio-Nagi-Concurrently	40.114	33.606	6.508	2.583	0.17 ± 0.02^c^
Bio-Nagi-Post	45.2	33.571	11.629	7.704	0.005 ± 0.00^d^
Bio4-Pre	39.558	33.571	5.987	2.062	0.24 ± 0.03^b^
Bio4-Concurrently	42.432	33.571	8.861	4.936	0.033 ± 0.004^c^
Bio4-Post	39.857	33.571	6.286	2.361	0.19 ± 0.02^b^
Control-Infected	41.5	33.571	7.929	4.004	0.06 ± 0.01^c^
Control-Healthy	37.496	33.571	3.925	0	1

Note: Actin was used as the reference gene, and the healthy control was used as the calibrator (ΔΔC_t_ = 0; fold change = 1). Values are expressed as mean ± SE. Different lowercase letters in the fold-change column indicate significant differences among treatments according to one-way ANOVA followed by Tukey’s HSD test at *p* ≤ 0.05; values sharing the same letter are not significantly different

### The relative expression levels of the PR3 (Chitinase) gene

**Fig. 9:**
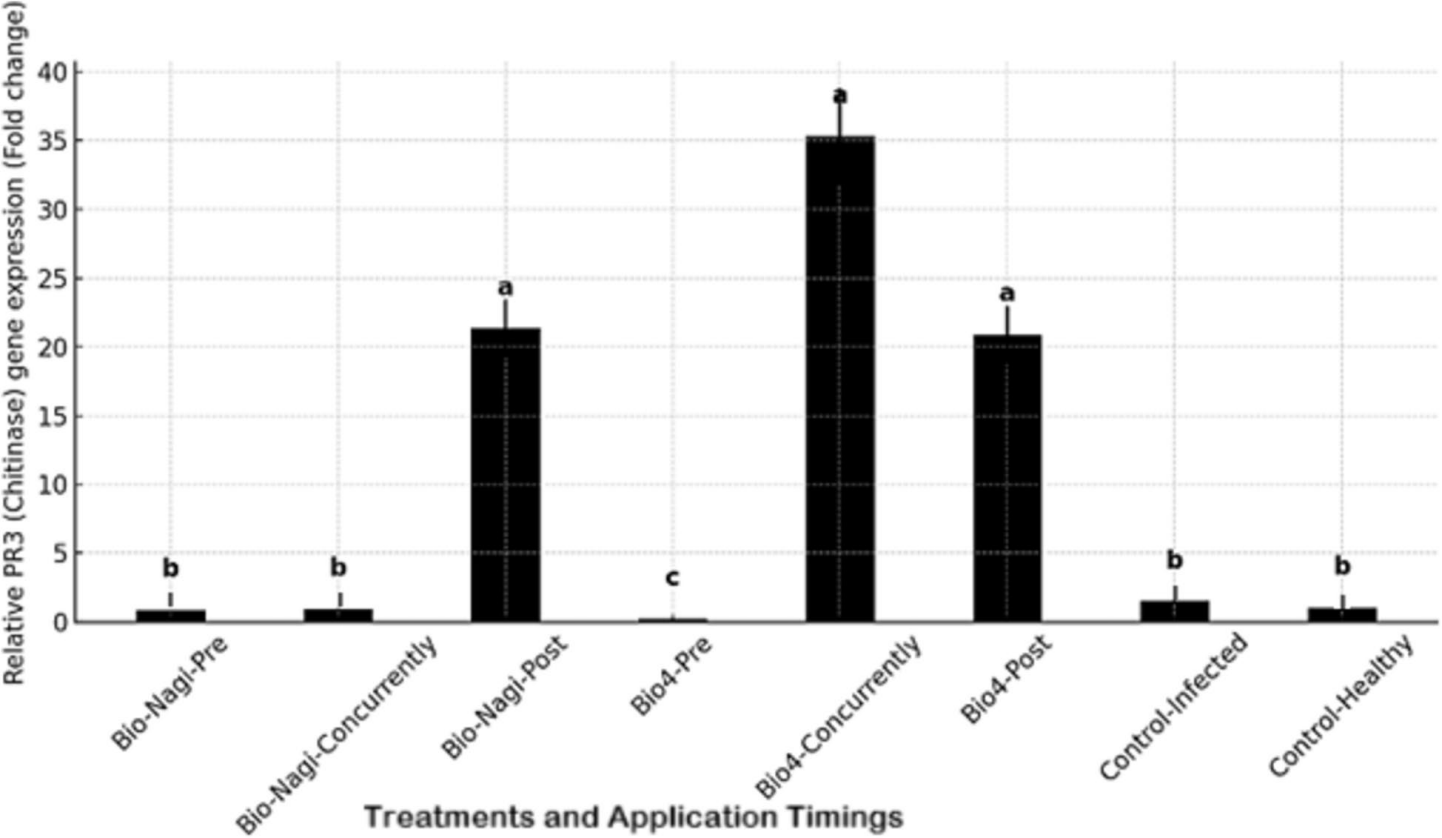
Relative expression of the PR3 (chitinase) gene in squash plants infected with ZYMV and treated with Bio-Nagi and Bio-4 at different application timings. Gene expression was quantified by qRT-PCR and normalized to *Actin* as the reference gene. Fold-change values were calculated using the 2^(-ΔΔCt) method relative to the healthy control. Treatments applied concurrently or post-inoculation with Bio-4 and Bio-Nagi exhibited significantly elevated PR3 expression levels. Error bars represent the standard error (SE), and different letters indicate statistically significant differences among treatments (*p* < 0.05). Full qRT-PCR data are provided in Additional file S1

The relative expression levels of the PR3 (Chitinase) gene in squash plants infected with Zucchini yellow mosaic virus (ZYMV) and treated with Bio-Nagi and Bio-4 at various application timings (pre-inoculation, concurrently, and post-inoculation) were assessed using the 2^(-ΔΔC_t_) method, normalized against healthy control plants. Significant variations (*p* < 0.05) were observed among treatments. The highest PR3 expression was significantly recorded in plants treated concurrently with Bio4, followed closely by treatments with Bio-Nagi post-inoculation ("Bio-Nagi-Post") and Bio4 post-inoculation ("Bio4-Post") (Table 4 and Fig. 9). These treatments exhibited notably elevated fold changes compared to the healthy control. In contrast, pre-inoculation treatments ("Bio-Nagi-Pre" and "Bio4-Pre") and the infected untreated control displayed significantly lower PR3 gene expression, indicating minimal induction of the host defense mechanism. These findings suggest that concurrent or post-inoculation application of Bio-Nagi and Bio-4 significantly activates chitinase-mediated defense responses, potentially providing effective control against ZYMV infection.

**Table 4:** ΔC_t_, ΔΔC_t_, and relative fold change (2⁻ΔΔC_t_) of PR3 (chitinase) gene expression in squash plants treated with Bio-Nagi and Bio-4 at different application timings relative to ZYMV inoculation

Treatment	C_t_ -PR3	C_t_-Actin	ΔC_t_	ΔΔC_t_	Fold Change (2^ ^−ΔΔCt^)
Bio-Nagi-Pre	41.5	33.536	7.964	0.294	0.82 ± 0.06^b^
Bio-Nagi-Concurrently	41.445	33.606	7.839	0.169	0.89 ± 0.07^b^
Bio-Nagi-Post	36.827	33.571	3.256	−4.414	21.32 ± 1.65^a^
Bio4-Pre	43.641	33.571	10.07	2.4	0.19 ± 0.03^c^
Bio4-Concurrently	36.101	33.571	2.53	−5.14	35.26 ± 2.10^a^
Bio4-Post	36.859	33.571	3.288	−4.382	20.85 ± 1.60^a^
Control-Infected	40.698	33.571	7.127	−0.543	1.46 ± 0.10^b^
Control-Healthy	41.241	33.571	7.67	0	1

Note: Actin served as the reference gene, and the healthy control was used as the calibrator (ΔΔC_t_ = 0; fold change = 1). Values are presented as mean ± SE. Different lowercase letters in the fold-change column indicate statistically significant differences among treatments according to one-way ANOVA followed by Tukey’s HSD test at *p* ≤ 0.05; treatments sharing the same letter are not significantly different

### The relative expression levels of the PR5 gene

**Fig. 10:**
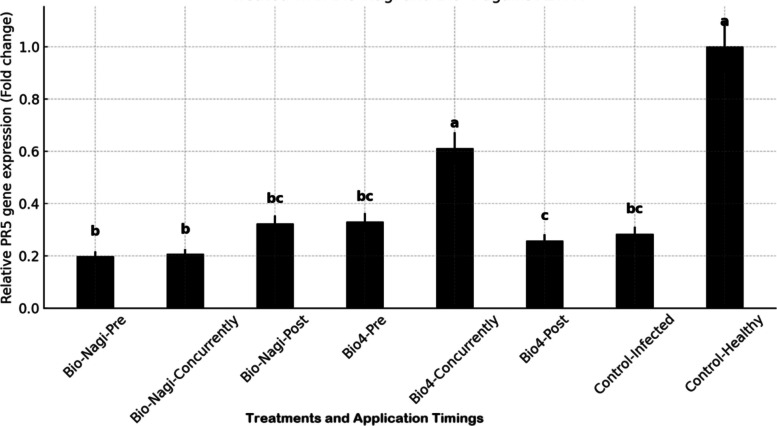
Relative expression of the PR5 (thaumatin-like protein) gene in squash plants infected with ZYMV and treated with Bio-Nagi and Bio-4 at pre-, concurrent-, and post-inoculation application timings. Gene expression was quantified by qRT-PCR, normalized to *Actin* as the reference gene, and calculated using the 2⁻ΔΔCt method relative to the healthy control. Error bars represent the standard error (SE). Different letters above the bars indicate statistically significant differences among treatments (*p* < 0.05). Full qRT-PCR data are provided in Additional file S1

The relative expression levels of the PR5 gene significantly varied among squash plants infected with Zucchini yellow mosaic virus (ZYMV) and treated with Bio-Nagi and Bio-4 at different application timings (pre-inoculation, concurrently, and post-inoculation), as assessed by the 2^(-ΔΔCt) method (*p* < 0.05). Plants treated concurrently with Bio4 ("Bio4-Concurrently") showed notably higher PR5 gene expression compared to other virus-infected treatments. Conversely, the lowest expression levels were observed in Bio-Nagi pre- and concurrent treatments ("Bio-Nagi-Pre," "Bio-Nagi-Concurrently"). Intermediate expression was recorded in Bio-Nagi post-inoculation ("Bio-Nagi-Post"), Bio4 pre- and post-inoculation treatments ("Bio4-Pre," "Bio4-Post"), and the infected untreated control (Table 5 and Fig. 10). The healthy control plants exhibited the highest baseline expression. These findings suggest that concurrent application of Bio4 notably enhances PR5 gene-mediated defense, potentially providing effective protection against ZYMV infection.

**Table 5:** ΔCt, ΔΔCt, and relative fold change (2⁻ΔΔCt) of PR5 (thaumatin-like protein) gene expression in squash plants treated with Bio-Nagi and Bio-4 at different application timings relative to ZYMV inoculation

Treatment	C_t_-PR5	C_t_-Actin	ΔC_t_	ΔΔC_t_	Fold Change (2^ ^−ΔΔC^_t_)
Bio-Nagi-Pre	34.81	33.536	1.274	2.336	0.20 ± 0.02^b^
Bio-Nagi-Concurrently	34.825	33.606	1.219	2.281	0.21 ± 0.02^b^
Bio-Nagi-Post	34.141	33.571	0.57	1.632	0.32 ± 0.03^bc^
Bio4-Pre	34.108	33.571	0.537	1.599	0.33 ± 0.03^bc^
Bio4-Concurrently	33.218	33.571	−0.353	0.709	0.61 ± 0.05^a^
Bio4-Post	34.468	33.571	0.897	1.959	0.26 ± 0.02^c^
Control-Infected	34.328	33.571	0.757	1.819	0.28 ± 0.02^bc^
Control-Healthy	32.509	33.571	−1.062	0	1

Note: Actin served as the reference gene, and the healthy control was used as the calibrator (ΔΔC_t_ = 0; fold change = 1). Values are presented as mean ± SE. Different lowercase letters in the fold-change column indicate statistically significant differences among treatments according to one-way ANOVA followed by Tukey’s HSD test at *p* ≤ 0.05; treatments sharing the same letter are not significantly different

## Discussion

Zucchini yellow mosaic virus (ZYMV) remains one of the most economically destructive viruses affecting cucurbit production worldwide. In this study, infected squash plants exhibited classical ZYMV symptoms-severe mosaic, vein banding, filiform leaf deformation, chlorotic mottling, and stunting-which are highly consistent with previously documented manifestations in Egypt and the Middle East [17]. Ultrastructural observations confirmed the cytopathological consequences of ZYMV infection, including chloroplast deformation, mitochondrial swelling, and nuclear disorganization. Such organelle degeneration aligns with the characteristic potyvirus-induced cytopathology described by Hammad et al. [22], who emphasized that virus-mediated disruption of cellular structures significantly contributes to symptom severity and impaired physiological performance.

Molecular diagnosis using CP-specific primers yielded a ~ 1221 bp coat protein (CP) fragment, confirming successful amplification of ZYMV. This finding reinforces the reliability of CP-based PCR assays, which remain the gold standard for ZYMV detection due to their high specificity and conservation across isolates [33]. Phylogenetic analysis revealed a tight clustering between the Egyptian isolate (ZYMV-EG,PV833355.1) and a German isolate (PP256252.1), with 99.67% identity and strong bootstrap support. This remarkable genetic similarity aligns with earlier reports indicating low global diversity within ZYMV populations due to seed transmission and the international movement of cucurbit germplasm [17, 27]. The strong conservation of the CP gene likely reflects evolutionary constraints associated with virion stability, aphid transmission efficiency, and systemic movement, all of which limit sequence variability and preserve functional domains [33].

Although molecular confirmation was successful, a methodological limitation of the study is the absence of quantitative viral load assessment. Endpoint RT-PCR, while sensitive, does not reflect viral accumulation levels; thus, treatment comparisons were based on symptom severity, antioxidant enzyme activity, ultrastructural restoration, and PR-gene expression rather than direct quantification of viral titers. Incorporating quantitative PCR or ELISA in future studies would strengthen conclusions and allow more precise assessment of viral suppression dynamics.

Gene expression analysis followed MIQE principles such as primer specificity and melt-curve validation. However, normalization relied on a single reference gene (β-actin). MIQE guidelines recommend validating multiple housekeeping genes for robust normalization. Despite this limitation, the expression patterns of PR1, PR3, and PR5 were biologically consistent and reproducible. Nonetheless, future studies should employ at least two reference genes to improve accuracy and reduce potential bias.

The study provides mechanistic insights into the antiviral effects of two newly developed Egyptian microbial formulations: Bio-Nagi, containing *Trichoderma asperellum*, and Bio-4, a four-species *Bacillus* consortium. By integrating symptom reduction with ultrastructural repair, antioxidant dynamics, and PR-gene expression, the study reveals that the two formulations activate distinct immune-signaling pathways and exert their strongest effects at different stages of infection.

Bio-Nagi exhibited its greatest antiviral efficacy when applied 48 h prior to inoculation, coinciding with a pronounced induction of PR1-a hallmark of salicylic acid (SA)-dependent systemic acquired resistance (SAR). This finding is strongly supported by previous reports showing that *Trichoderma* spp. are highly effective at priming SA signaling and activating SAR-mediated antiviral responses [10, 25]. The substantial PR1 upregulation observed here parallels earlier findings where *Trichoderma*-based biocontrol agents enhanced SA-related defense mechanisms against ZYMV and other potyviruses [1, 14, 16, 34]. These results collectively indicate that Bio-Nagi acts primarily as a preventive SAR-inducing agent, requiring pre-infection activation to maximize antiviral protection.

In contrast, Bio-4 demonstrated superior antiviral activity during concurrent and post-inoculation treatments, matching the strongest induction of PR3 (chitinase) and PR5 (thaumatin-like protein)-classic markers of jasmonic acid (JA) and ethylene (ET)-regulated induced systemic resistance (ISR). This pathway is typically activated by *Bacillus* species and other PGPR through mechanisms such as siderophore production, volatile organic compounds, nutrient solubilization, and microbial RNases [11, 21, 36, 43]. The robust PR3 induction observed during concurrent Bio-4 application aligns with the concept that ISR is most effective when triggered in the presence of pathogen cues. These results suggest that Bio-4 acts mainly as a curative ISR-inducing formulation, particularly effective once infection has begun.

Ultrastructural data supported these molecular findings: Bio-4 treatments restored chloroplast structure, alleviated mitochondrial swelling, and improved nuclear organization more effectively than Bio-Nagi, especially during post-inoculation. This superior restorative effect likely stems from enhanced ROS detoxification and PR-protein activity. Indeed, Bio-4 produced the strongest increase in superoxide dismutase and peroxidase activities-enzymes central to oxidative stress mitigation-consistent with the known antioxidant-modulating activity of *Bacillus*-mediated ISR [7, 31, 32]. Conversely, Bio-Nagi induced more modest increases in antioxidant enzymes, reflecting its reliance on SAR and PR1 pathways rather than ROS-scavenging mechanisms.

The qRT-PCR results further differentiated the timing-dependent action of each formulation. Bio-Nagi produced a strong PR1 response only when applied before inoculation, consistent with SAR’s requirement for early activation. Bio-4, however, produced an exceptionally high PR3 response during concurrent treatment, confirming that ISR is strongest during or shortly after pathogen challenge [20, 43]. These timing-dependent patterns clearly indicate that SAR and ISR are engaged at different stages of infection, and that microbial treatments must be aligned with the correct temporal window to maximize antiviral defense.

Compared with previous studies on microbial biocontrol of ZYMV, the present research provides a significantly more integrated mechanistic framework. Earlier studies often focused on symptom reduction or biochemical changes without linking these outcomes to specific hormonal pathways or ultrastructural responses. Ghanem et al. [17] and Aseel et al. [9] demonstrated reduced virus severity using *Streptomyces* or *Trichoderma* species but did not elucidate pathway-specific signaling or cellular repair. Even recent comprehensive reviews [5, 30] highlight the scarcity of studies combining gene expression, biochemical markers, and ultrastructural evidence. Here, the integration of disease severity, antioxidant responses, cytological restoration, and differential PR-gene activation provides one of the most complete mechanistic assessments yet reported for ZYMV biocontrol.

Moreover, the exploration of application timing represents a methodological advance. Few studies have compared pre-, concurrent-, and post-inoculation treatments, despite timing being crucial for determining whether SAR or ISR dominates. The finding that Bio-Nagi is most effective before infection, whereas Bio-4 is more effective during and after infection, adds a practical dimension to microbial application strategies in cucurbit production systems.

Finally, this work aligns with emerging evidence that *Bacillus* consortia surpass single-strain biocontrol agents because of their broader metabolic potential and complementary modes of action [20, 39]. Bio-4 consistently exceeded Bio-Nagi across biochemical, ultrastructural, and molecular parameters, strengthening the case for multi-strain formulations in antiviral management.

Overall, this study makes a significant contribution by providing a multilayered mechanistic explanation of how Bio-Nagi and Bio-4 differentially activate SAR and ISR pathways against ZYMV, revealing clear timing-dependent distinctions and offering a scientific basis for optimizing microbial biocontrol strategies in squash. The integration of molecular, physiological, cytological, and biochemical evidence establishes one of the most comprehensive mechanistic frameworks to date for antiviral microbial control in cucurbits.

Although the present study was conducted under controlled greenhouse conditions, the strong antiviral responses induced by Bio-Nagi and Bio-4-particularly their ability to activate SAR and ISR pathways, enhance antioxidant enzyme activities, and restore cellular integrity-indicate promising potential for field application. Both formulations consist of naturally occurring microbial agents known for their environmental stability and adaptability, suggesting that they can withstand variable field conditions. In addition, Bacillus-based consortia such as Bio-4 are well documented for their resilience to temperature fluctuations, UV exposure, and soil moisture variability, which supports their suitability for open-field deployment. Trichoderma-based formulations such as Bio-Nagi also demonstrate strong rhizosphere colonization capacity and long-lasting association with plant roots, further enhancing their field relevance. Future multi-location field trials under natural disease pressure will be essential to validate the efficacy observed in greenhouse experiments and to determine their practical integration into commercial squash production systems.

Future studies should include large-scale multi-location field trials to validate treatment efficacy under natural disease pressure and environmental fluctuations. Incorporating additional squash cultivars with differing genetic backgrounds would also help assess genotype × treatment interactions. Comprehensive transcriptomic or RNA-seq analysis, combined with quantitative viral load measurements, would provide deeper mechanistic insights into defense pathway activation. Long-term evaluations of yield performance, microbial colonization dynamics, and metabolomic shifts following Bio-4 or Bio-Nagi applications will further strengthen their development as sustainable biocontrol strategies against ZYMV in commercial production systems.

## Conclusions

This study demonstrates that Bio-Nagi (*Trichoderma asperellum*) and Bio-4 (a *Bacillus* consortium) can enhance squash tolerance to ZYMV through activation of distinct defense pathways rather than through direct antiviral inhibition. The differential induction of PR1, PR3, and PR5 suggests that these microbial agents modulate both ISR- and SAR-related responses, with Bio-4 strongly stimulating chitinase-associated defense (PR3) during and after infection, while Bio-Nagi preferentially primed PR1-linked signaling when applied prior to inoculation. These mechanistic patterns highlight the importance of application timing in shaping the nature and intensity of induced resistance. From an applied perspective, microbial formulations such as Bio-4 and Bio-Nagi show promise as sustainable components of integrated viral disease management programs, particularly where chemical antiviral options are limited. Their ability to enhance physiological resilience, prime host defense, and mitigate symptom development under infection pressure positions them as potential field-applicable biocontrol tools. Future work integrating quantitative viral load assessments, broader defense-gene profiling, hormone signaling analyses, and multi-season field trials will be crucial to validate their robustness and inform effective deployment strategies in commercial production systems.

## Supplementary Information

Supplementary Material 1.

Supplementary Material 2.

## Data Availability

All data generated or analyzed during this study are included in this published article. The ZYMV isolate sequence has been deposited in GenBank under accession number PV833355. Additional datasets are available from the corresponding author upon reasonable request.

## Abbreviations

ZYMV: Zucchini yellow mosaic virus
RT-PCR: Reverse transcription polymerase chain reaction
qRT-PCR: Quantitative real-time polymerase chain reaction
TEM: Transmission electron microscopy
SOD: Superoxide dismutase
POD: Peroxidase
PR genes: Pathogenesis-related genes
MIQE: Minimum Information for Publication of Quantitative Real-Time PCR Experiments
