# Supplementary material for: Molecular and enzymatic insights into biocontrol-mediated resistance against Zucchini yellow mosaic virus in squash (Cucurbita pepo L.)
Source: BMC Microbiol. 2026 Jun 30;26:577. doi: 10.1186/s12866-026-05161-x (PMC13321636; doi:10.1186/s12866-026-05161-x)

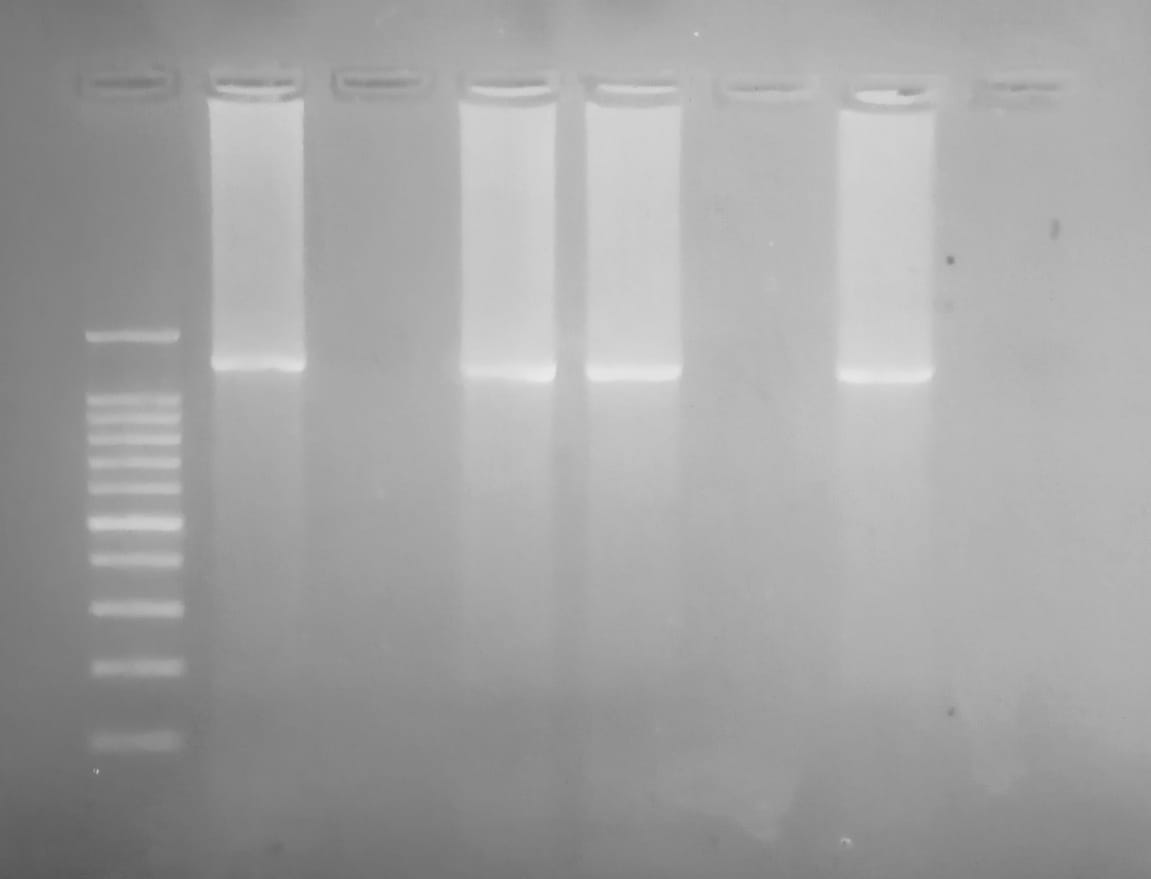


M PC NC L1 L2 L3 L4


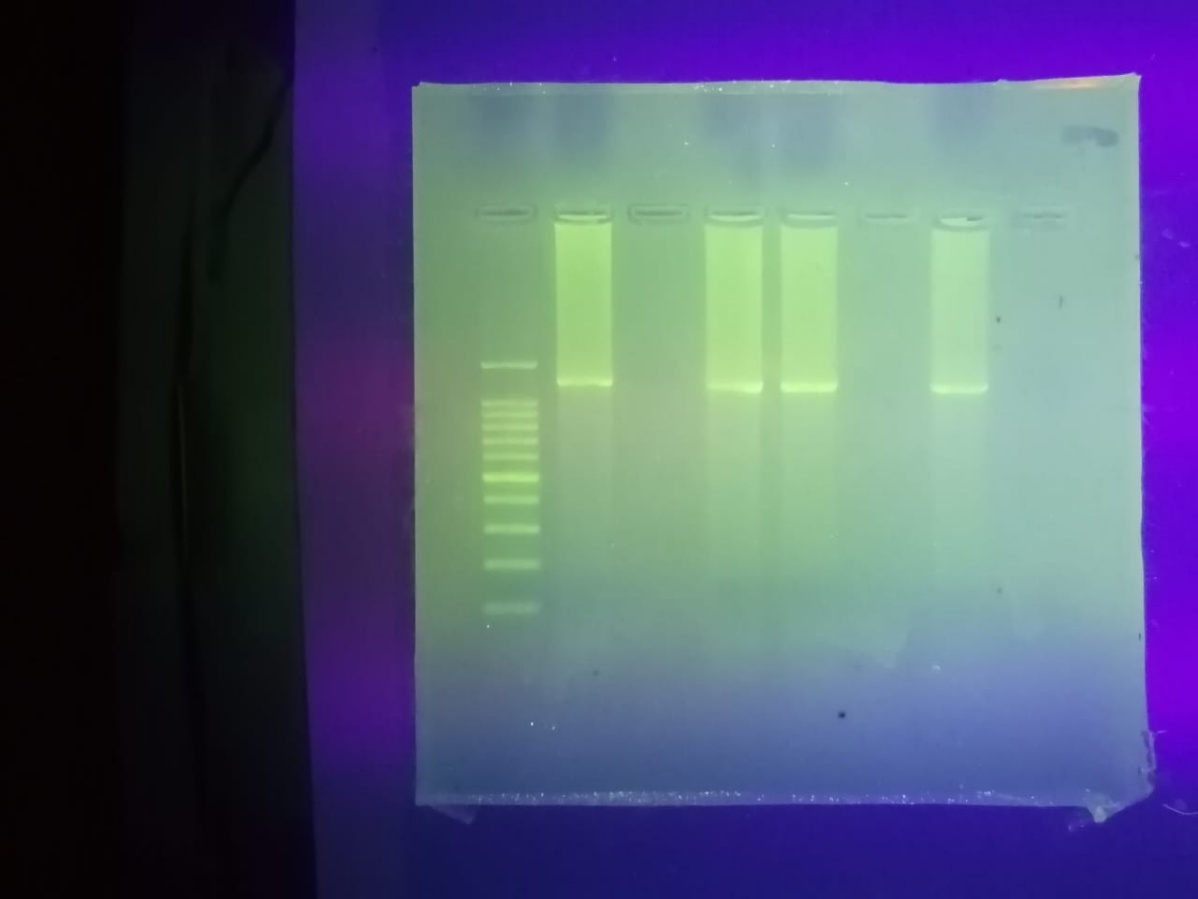


M PC NC L1 L2 L3 L4


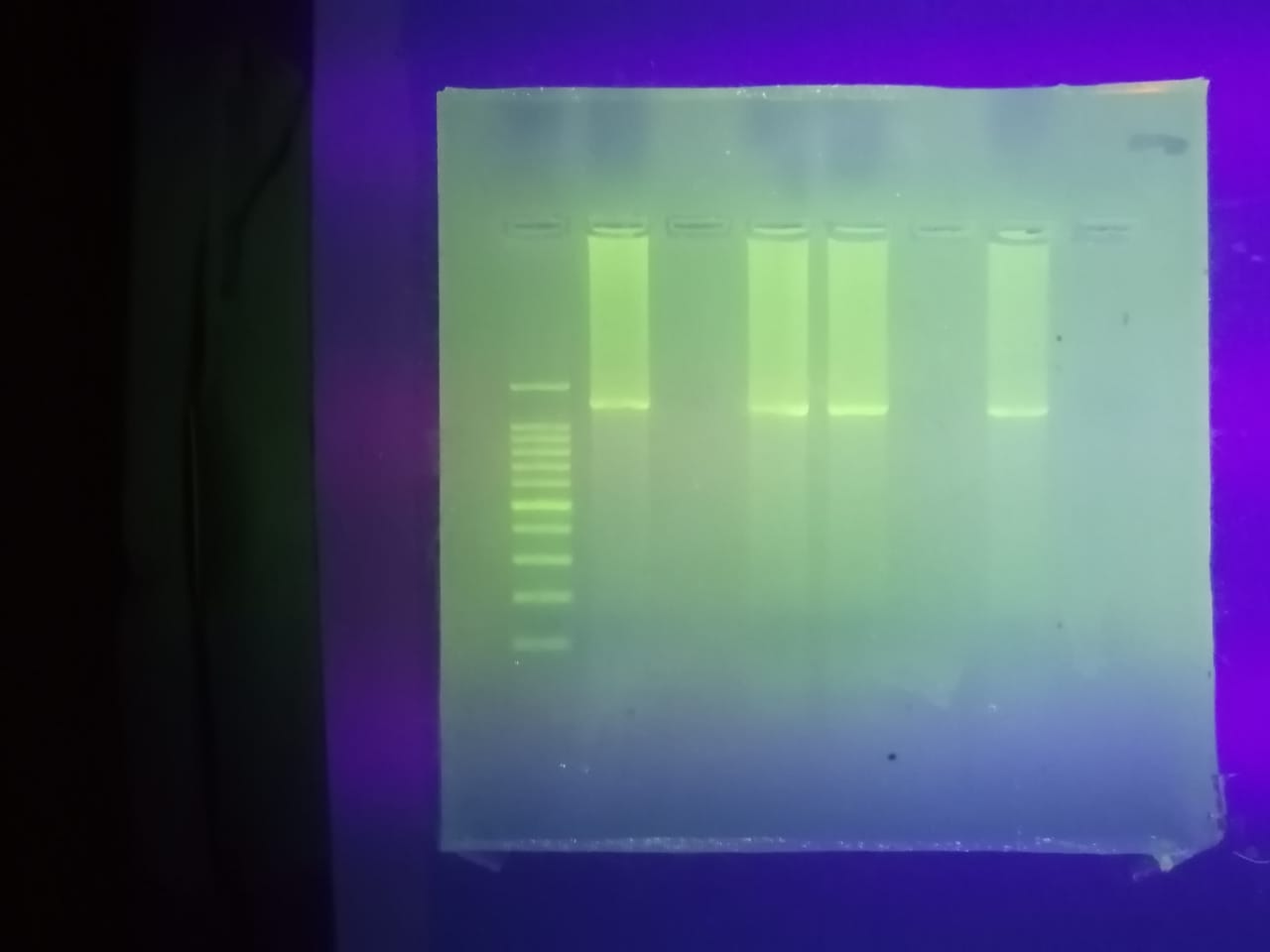


M PC NC L1 L2 L3 L4

Fig. S2A. RT-PCR amplification of *Zucchini yellow mosaic virus* (ZYMV) coat protein gene showing specific amplicons of approximately 1221 bp from infected squash plants. Lane M: 100 bp DNA ladder; lanes PC, NC, L1, L2, L3, L4: samples from positive control (PC), Negative control (NC), L1, L2 & L4 infected squash plants, L3: healthy squash plants.

β-actin


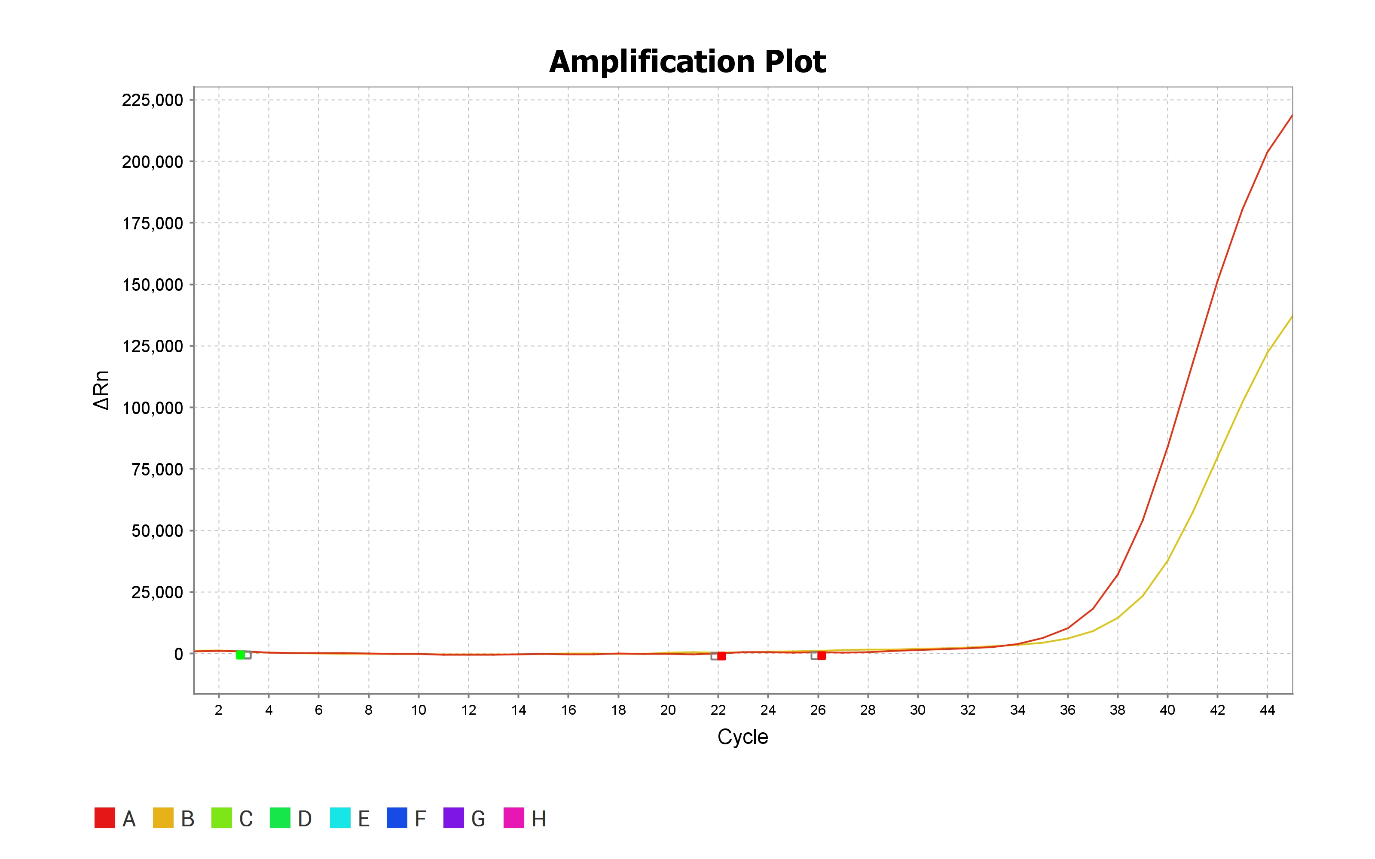


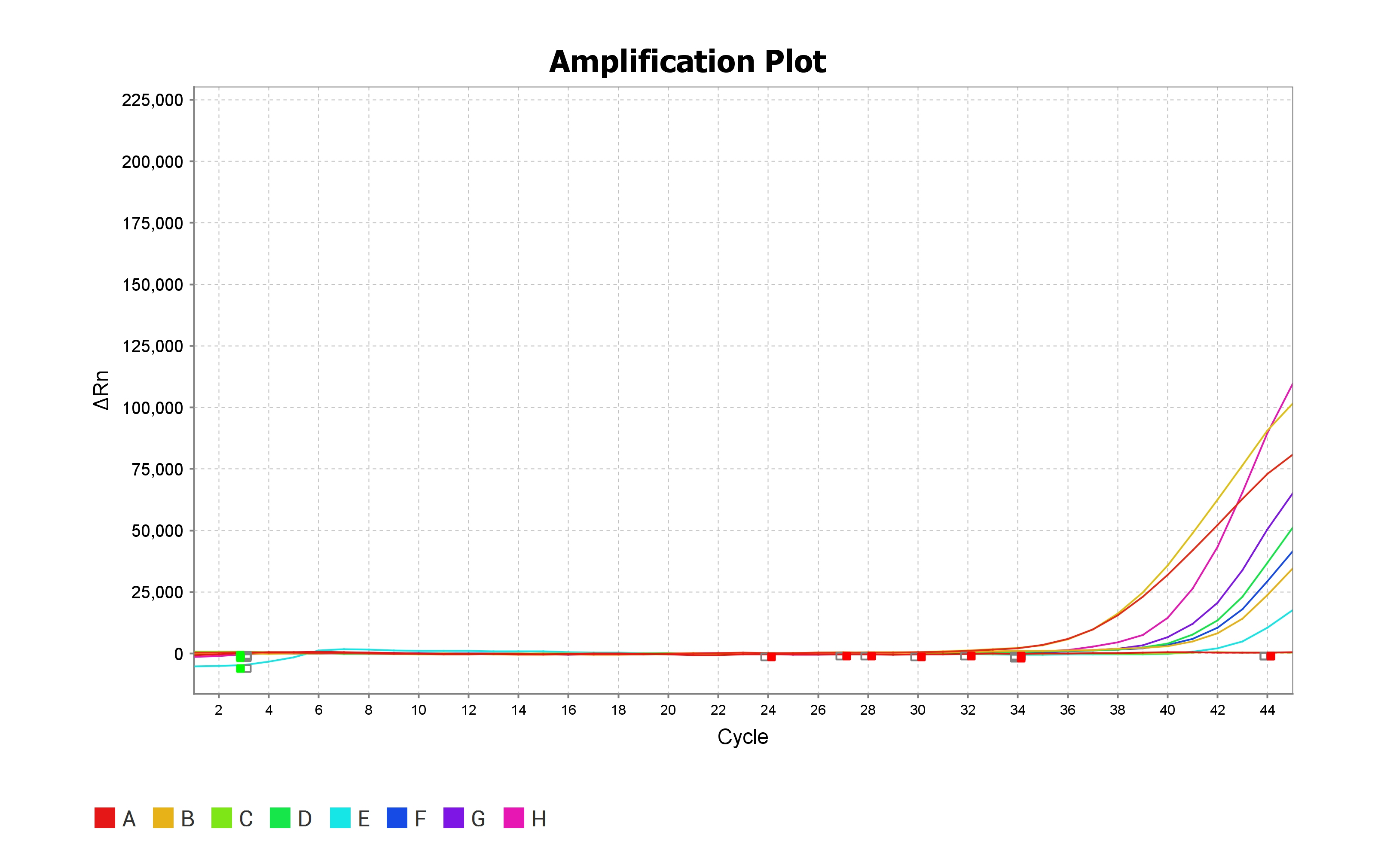


Pathogenesis-related protein-1 (PR1)

Chitinase (PR3)


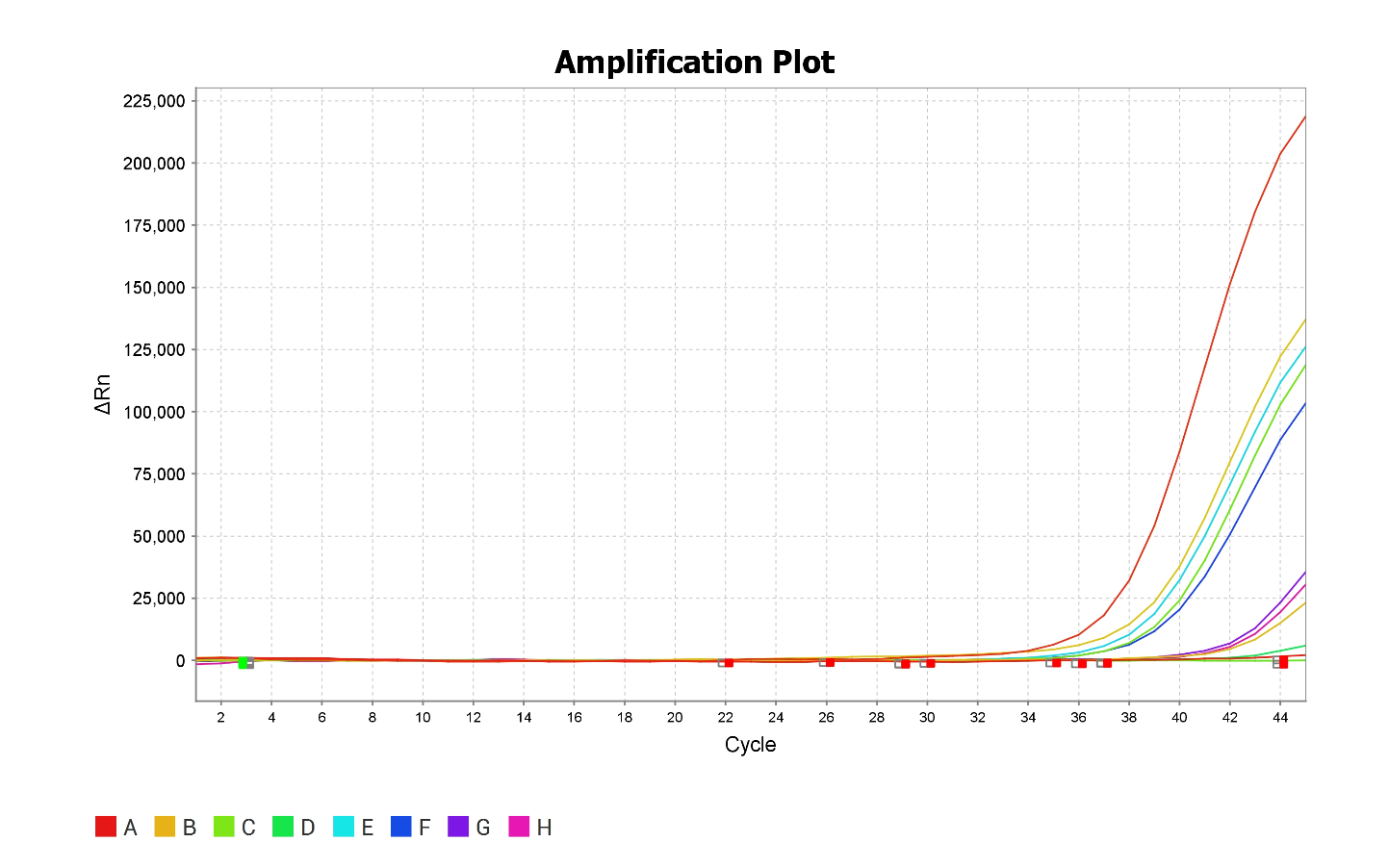


Thaumatin-like protein (PR5)


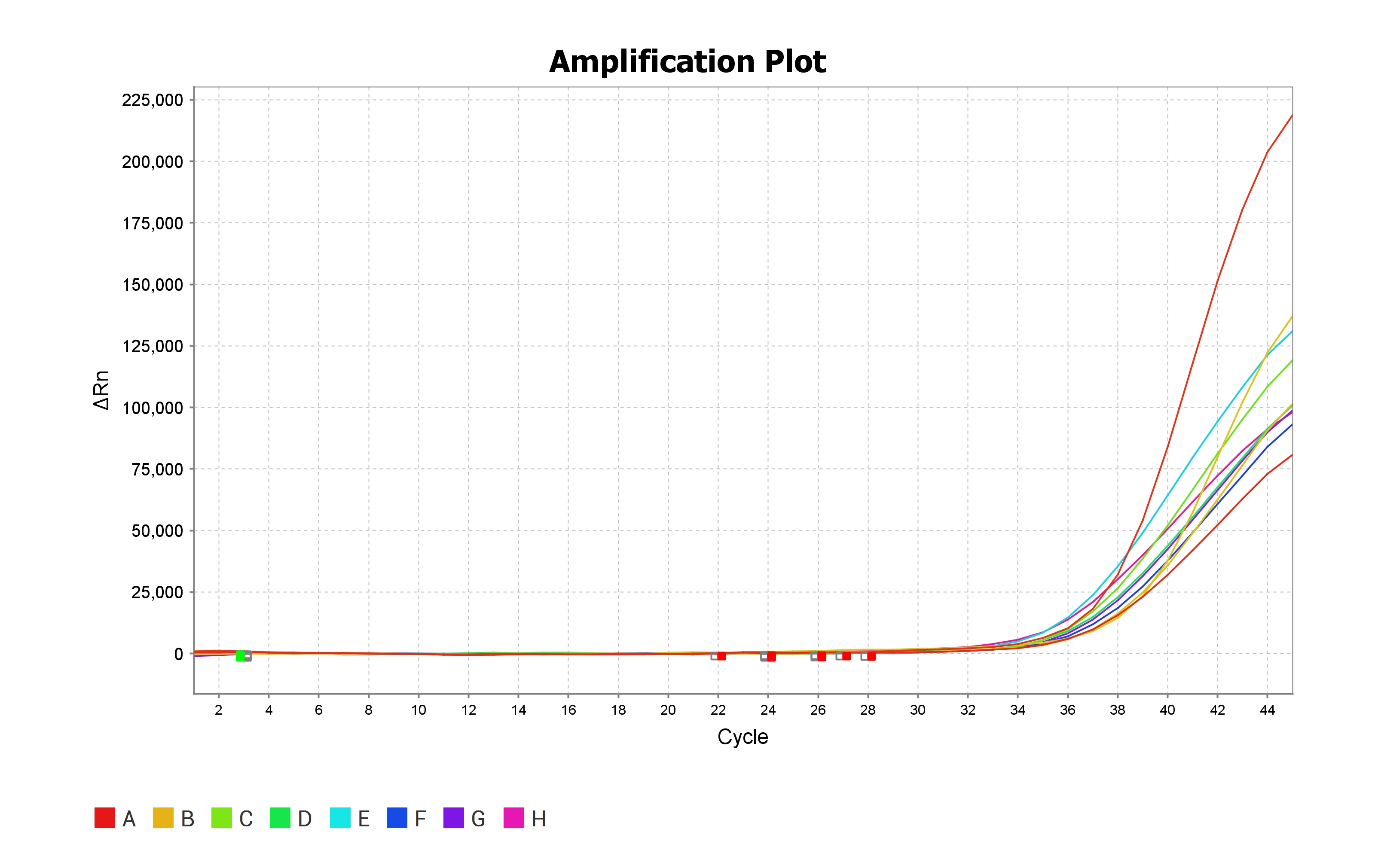


Amplification plot of PR1, PR3, PR5 and B-actin


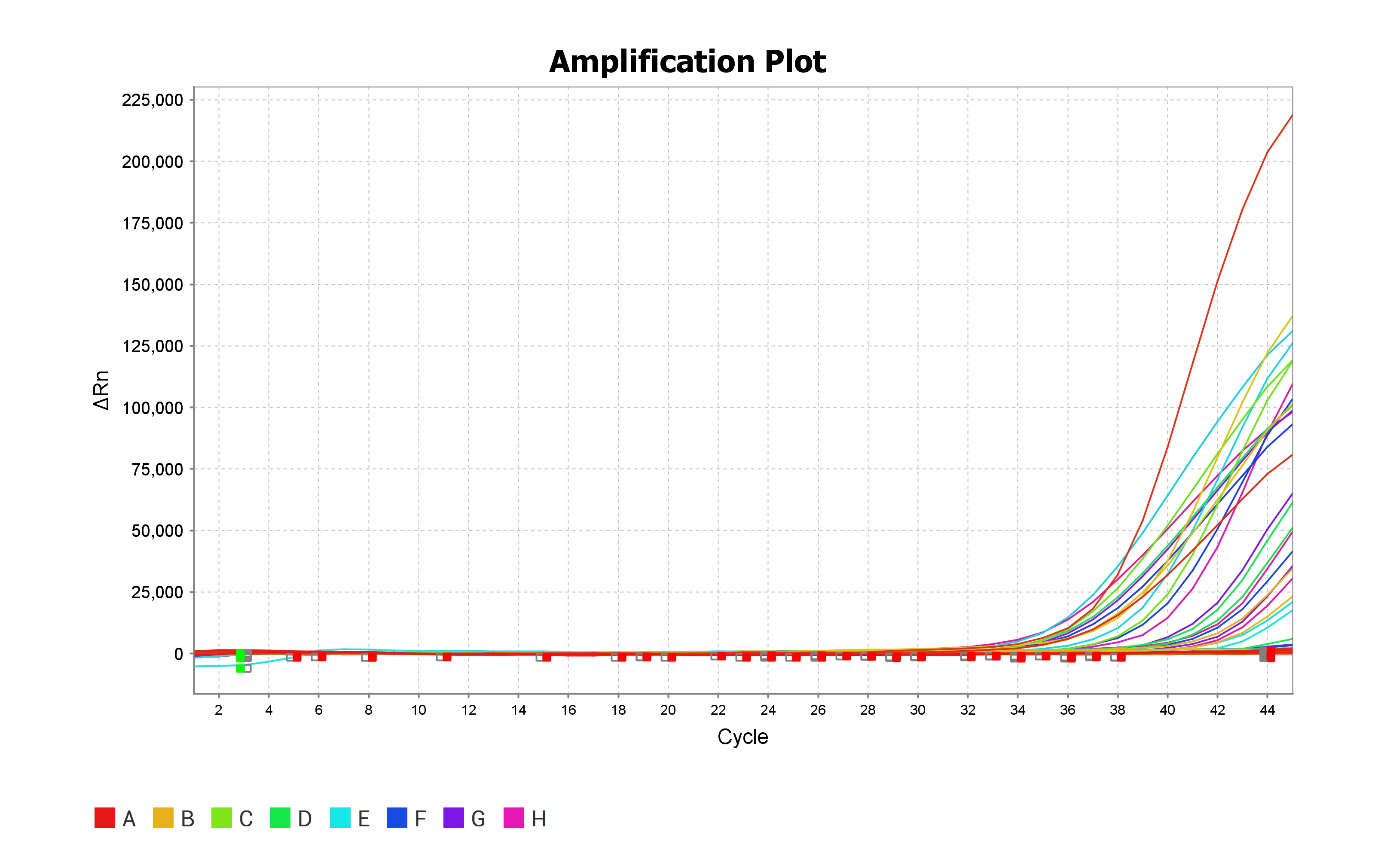

Supplement: Supplementary file 1 — Supplementary Material 1. [file 12866_2026_5161_MOESM1_ESM.docx]
