# Supplementary material for: Molecular and enzymatic insights into biocontrol-mediated resistance against Zucchini yellow mosaic virus in squash (Cucurbita pepo L.)
Source: BMC Microbiol. 2026 Jun 30;26:577. doi: 10.1186/s12866-026-05161-x (PMC13321636; doi:10.1186/s12866-026-05161-x)
Supplement: Supplementary file 2 — Supplementary Material 2. [file 12866_2026_5161_MOESM2_ESM.docx]

**Table S1**. Primer sequences used for RT-PCR and qRT-PCR amplification of ZYMV-CP and defense-related genes in *Cucurbita pepo* cv. Eskandarani.

| Gene | Primer Name | Sequence (5′→3′) |
| --- | --- | --- |
| ZYMV-CP | ZYU-F | GCTCCATACATAGCTGAGACAGC |
|  | ZYD-R | TAGGCTTGCAAACGGAGGTCTAATC |
| Pathogenesis-related protein-1 | PR-1F | GTTCCTCCCTTGGCCACCTTC |
|  | PR-1R | TATGACCCCCAACGAAGATG |
| Chitinase | PR-3F | ATGGAGCATTGTGCCCTAAC |
|  | PR-3R | TCCTGACCATGACCACCCA |
| Thaumatin-like protein | PR-5F | AATTGCAATTTTTAATGGGTGC |
|  | PR-5R | TAGCAGACCGTTTAGATGC |
| β-actin | β-actin-F | TGGCAATGAGCGGTTCAGTC |
|  | β-actin-R | ACTCAATCCCAAAGCCAACAGAGA |

ZYMV-CP: Zucchini yellow mosaic virus coat protein, β-actin: Beta-actin, a housekeeping gene used as an internal control for normalization in gene expression analysis.
